# Multifunctional Injectable Bioadhesive with Toll-like Receptor 4 and Myeloid Differentiation Factor 2 Antagonistic Anti-inflammatory Potential for Periodontal Regeneration

**DOI:** 10.1021/acsnano.4c15922

**Published:** 2025-02-14

**Authors:** Shuting Gao, Huihua Li, Zekun Li, Hong Wang, Xinyue Li, Shengyan Yang, Lin Huang, Baoping Zhang, Kailiang Zhang, James Kit Hon Tsoi, Jian He, Waruna Lakmal Dissanayaka

**Affiliations:** † Applied Oral Sciences & Community Dental Care, Faculty of Dentistry, The University of Hong Kong, Hong Kong, Hong Kong SAR 999077, China; ‡ Department of Chemistry, Faculty of Science, The University of Hong Kong, Hong Kong, Hong Kong SAR 999077, China; § State Key Laboratory of Applied Organic Chemistry and Key Laboratory of Nonferrous Metal Chemistry and Resources Utilization of Gansu Province, Lanzhou University, Lanzhou 730000, China; ∥ Department of Stomatology Lanzhou University, Lanzhou University, Lanzhou 730000, China

**Keywords:** periodontal regeneration, antagonist, multifunctional hydrogel, anti-inflammation, xylitol, bioadhesive, injectable hydrogels

## Abstract

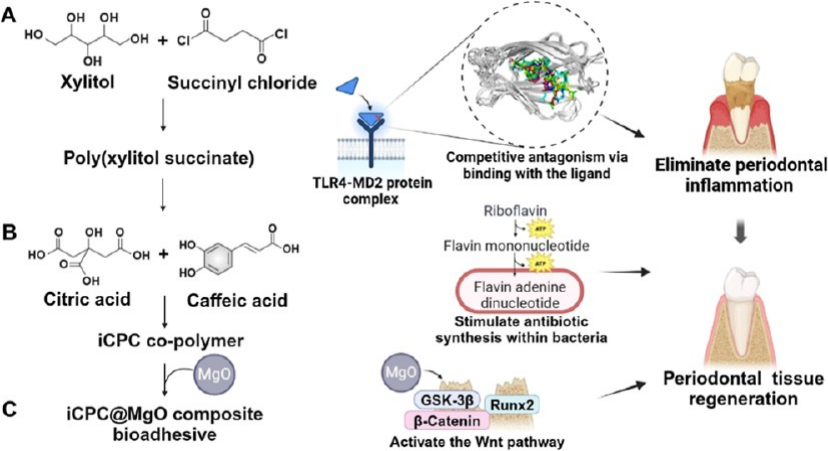

Effectively addressing inflammation in periodontitis is challenging as conventional injectable hydrogels typically require the addition of drugs to provide sufficient anti-inflammatory effects. To overcome this limitation, we developed a multifunctional injectable hydrogel with inherent properties that antagonize the Toll-like receptor 4 and myeloid differentiation factor 2 complex (TLR4-MD2). This hydrogel allows for direct inhibition of inflammatory pathways without the need for additional drugs. We identified xylitol, caffeic acid, and citric acid as natural materials that effectively meet biological needs for anti-inflammatory and antibacterial effects as well as support bone regeneration. With this in mind, we developed a caffeic-acid-modified poly(xylitol succinate) (PXS)-based iCPC@MgO composite hydrogel and tested its potential application for periodontal regeneration. The iCPC@MgO hydrogel demonstrated rapid wet tissue adhesion and injectability, which are ascribed to incorporating catechol groups derived from caffeic acid. Intriguingly, the PXS polymer used for synthesizing the hydrogel was found to possess anti-inflammatory properties and act as an antagonist for the TLR4-MD2 complex. This hydrogel also exhibited outstanding antibacterial efficiency against *Porphyromonas gingivalis* and *Aggregatibacter actinomycetemcomitans* by stimulating antibiotic synthesis within bacteria and disrupting bacterial cell walls. In a periodontitis mouse model, the iCPC@MgO hydrogel demonstrated the therapeutic potential of reducing inflammatory factors, inhibiting dominant periodontitis-associated bacteria, and maintaining subgingival microbiota balance in addition to the regenerative effects. These properties, combined with their ecofriendly nature, firmly established the iCPC@MgO hydrogel as a highly promising option for use in periodontitis therapy as well as in tissue healing, repair, and regeneration in various other inflammatory conditions.

Periodontal disease (PD) is the 11th most prevalent disease among adults, affecting 20–50% of the global adult population.^1^ Periodontitis begins with oral bacterial dysbiosis and involves persistent inflammation in periodontal tissues, resulting in damage to soft and hard tissues and ultimately tooth loss. Current clinical treatments for periodontitis, such as flap debridement and tissue regeneration strategies, often struggle to maintain a stable anti-inflammatory environment.^2^ Effective anti-inflammatory functionality is crucial to control ongoing inflammation and support long-term tissue repair, which are essential for improving periodontitis outcomes.

In periodontitis, dysregulated chronic or local inflammation arises from damaged cells or pathogens, leading to the recruitment of immune cells and the production of pro-inflammatory cytokines such as tumor necrosis factor (TNF), interleukin-1 (IL-1), and IL-6.^3^ Such a dysregulated immune response can trigger a detrimental cascade, marked by activated osteoclasts and impaired tissue healing processes, ultimately leading to unsatisfactory therapeutic outcomes.^4^ To address this issue, many researchers have concentrated on improving polymer-based hydrogels to achieve anti-inflammatory effects, often using drug delivery methods.^5^ Several well-known anti-inflammatory agents, such as small molecular drugs, biomacromolecules (including nucleic acids, peptides, and proteins), and nanoparticles, have been investigated for their potential use.^6,7^ However, these agents possess certain limitations that hinder their efficacy in delivering anti-inflammatory treatments for periodontitis. For instance, the existence of nonpolar or hydrophobic regions in most nonsteroidal anti-inflammatory drugs (NSAIDs) (such as meloxicam and celecoxib) leads to their low solubility, consequently reducing their effectiveness as therapeutic agents.^8^ The biomacromolecule treatment, including the glycogen synthase kinase 3 beta (GSK3β) or TNFα inhibitors, IL-10, and sinensetin, have been developed as carriers for their anti-inflammatory properties.^9^ However, several drawbacks have specifically limited their ultimate clinical translation, including low in vitro permeability due to cell membrane resistance and easy degradation by abundant blood enzymes such as proteases, DNases, and RNases.^10^ As a result, there are still challenges in achieving anti-inflammatory functionality in polymer-based hydrogels. One significant factor is the role of the Toll-like receptor 4 (TLR4) and myeloid differentiation factor 2 (MD2) complex, which is crucial in regulating the inflammatory response in periodontitis.^11^ When activated, this complex triggers a signaling cascade that releases pro-inflammatory cytokines, contributing to excessive inflammation.^12^ Therefore, targeting the TLR4-MD2 complex presents a promising strategy for developing effective anti-inflammatory therapies. Instead of focusing only on the loaded agents, these concerns could be addressed by using and modifying polymers with inherent anti-inflammatory properties to antagonize the TLR4-MD2 complex. This approach reduces the need for additional agents and their potential side effects, optimizing the performance and applicability of polymers in hydrogel applications.

Xylitol is a natural compound that has been reported for its anti-inflammatory properties and could serve as a raw source for synthesizing polymers.^13^ Many formulations of xylitol, including diverse polyesters, e.g., xylitol-sebacic acid,^14^ xylitol-glutamic acid,^15^ and xylitol-succinate acid (PXS),^16^ have been explored as candidates to synthesize polymers. Xylitol-based polymers exhibit excellent biodegradability, biocompatibility, and low cytotoxicity in vitro and in vivo compared with synthetic polymers like poly(l-lactic-co-glycolic acid) (PLGA).^17^ However, little has been done to develop a xylitol-based bioadhesive hydrogel so far. More importantly, xylitol-containing products are also well known in dentistry for reducing dental plaque accumulation by inhibiting the growth of pathogenic bacteria, such as *Porphyromonas gingivalis* and *Actinobacteria*, which are linked to periodontitis. Considering these benefits, we aimed to develop a xylitol-based polymer as the foundational material for an adhesive hydrogel.

Strong adhesion to the root surface and surrounding gingival soft tissues in the wet state is essential for improving therapeutic efficacy, which can be achieved through polymer-based bioadhesive hydrogels. The most commonly reported bioadhesives currently are mussel-inspired, containing the catecholic amino acid 3,4-dihydroxyphenylalanine (DOPA). This compound and its catechol derivatives have numerous polymeric applications as adhesive reinforcers.^18,19^ However, due to neurotoxicity, it might not be the optimal adhesive component for application in biological tissue regeneration.^20^ Caffeic acid (3,4-dihydroxy-cinnamic acid), on the other hand, is a natural compound found in various plant sources, including coffee, fruits, and vegetables. It is an example of a catecholic organic compound that can potentially serve as a suitable adhesive polymer. Beyond that, the outstanding properties of caffeic acid include anti-inflammatory effects on periodontal tissue,^21^ antibacterial activity,^22^ antioxidant properties, and prevention of a variety of diseases by metabolic interference or apoptosis induction.^23^ Adding citric acid to the polymer increases the abundance of carboxyl groups, enabling the pH of its aqueous solution to approach 2.5.^24^ As a result, it acts as a gentle etchant on root dentin and surrounding alveolar bone, causing partial demineralization and exposure of collagen fibers.^25^ This process facilitates chemical anchoring and the formation of a hybrid layer, which allows for the development of a self-etch adhesive.

Cross-linking fabrication is a successful strategy for different kinds of polymer to form a mechanically enhanced hydrogel. Recently, growing interest has been in applying magnesium ions (Mg^2+^) in polymeric hydrogels. Researchers have chosen different polymer carriers coordinated with Mg^2+^, including polyacrylamide (PAM), polydopamine (PDA), and chitosan, to develop composite hydrogel systems.^26^ It has been dedicated to improving the mechanical properties of hydrogels or shortening the gelation time as one of the cross-linkers.^27^ In fact, the ability of magnesium oxide (MgO) to promote bone formation can help us achieve the goal of achieving multifunctional hydrogels. Researchers have conducted a systematic dissection of the central role of magnesium in bone regeneration in triggering macrophage-mediated osteogenesis by TRPM7 and activating MAPK/ERK or Wnt/β-catenin signaling pathway.^28^ The enhanced bone regeneration of Mg^2+^ was reflected in the increased overall rate of seeded calcium phosphate crystallization and the subsequent growth of hydroxyapatite (HA),^29^ and its superior osteogenic capacity has been shown in many reports.^30^

Herein, we aimed to address the inflammatory challenge in periodontitis by developing an injectable xylitol-based polymer hydrogel with tissue adhesive characteristics (Scheme 1). We synthesized poly(xylitol succinate) (PXS) through an esterification process by reacting xylitol with succinic acid. Notably, the PXS polymer demonstrated antagonistic binding to TLR4-MD2, surpassing the performance of Eritoran in terms of anti-inflammatory efficacy. By continuously esterifying caffeic acid and citric acid, we successfully incorporated these components into an iCPC copolymer, enhancing its adhesive properties. The final hydrogel was fabricated using MgO particles cross-linked with the iCPC copolymer, enabling sustained magnesium release, which contributes to osteogenic differentiation functionality. Additionally, the natural raw materials employed in the synthesis impart advanced antibacterial capabilities to the iCPC@MgO composite hydrogel. This innovative hydrogel effectively addresses the limitations of existing synthetic-polymer-based formulations by integrating potent anti-inflammatory and antibacterial properties, making it a promising candidate for the clinical management of periodontitis and other inflammatory conditions.

**Scheme 1:**
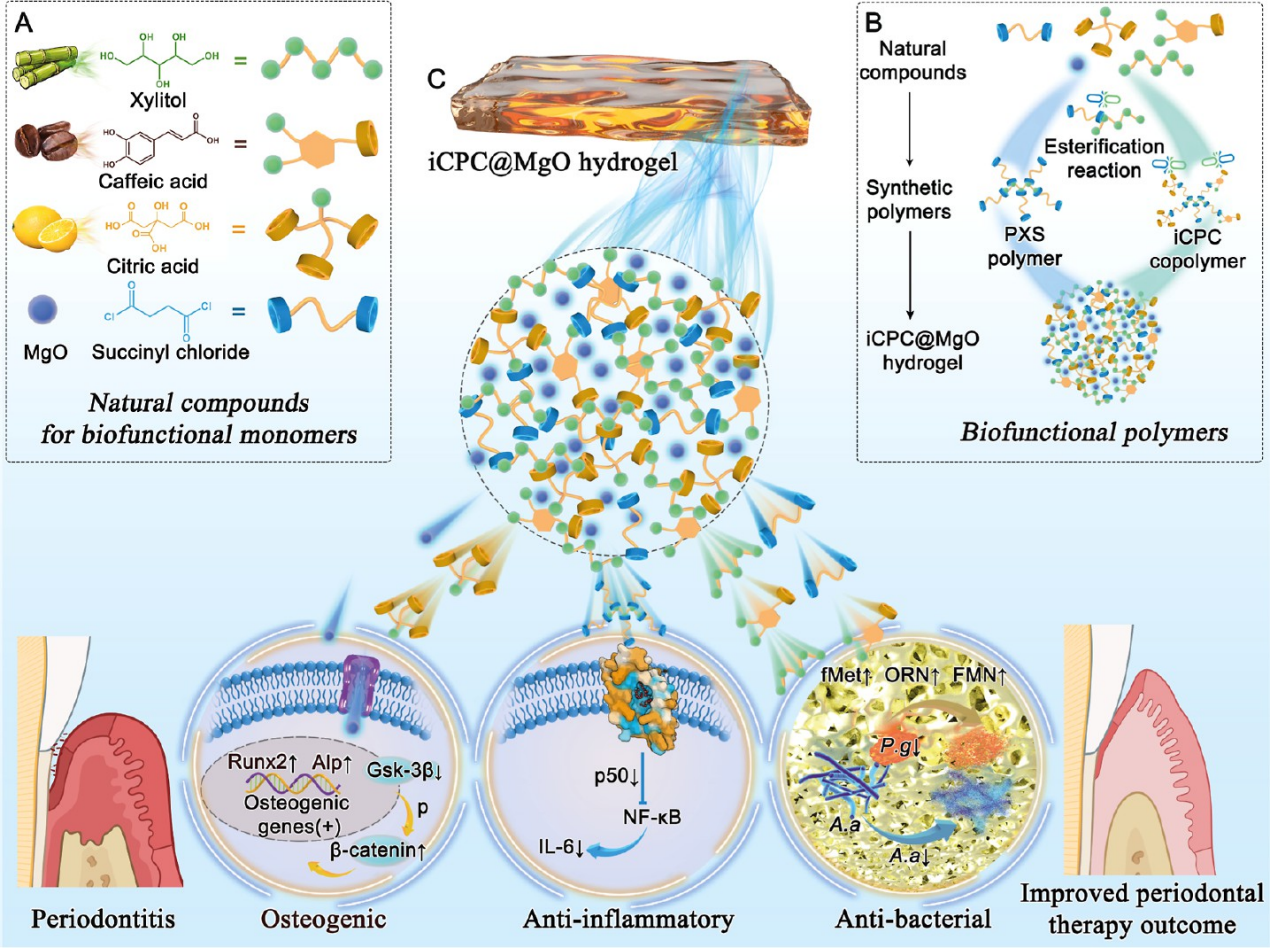
Schematic Diagram Illustrating the Synthesis of a Natural Resource-Based Copolymer in Developing an Injectable iCPC@MgO Multifunctional Hydrogel for Enhancing In Situ Periodontitis Treatment Outcomes. (A) Natural Raw Materials Used as Monomers in Polymer Synthesis. (B) Organization of the Natural Compounds into PXS and iCPC Polymers through an Esterification Reaction. (C) The iCPC@MgO Composite Hydrogel Has Components That Exert Anti-inflammatory, Antibacterial, and Osteogenic Effects to Improve Treatment Outcomes in Periodontitis

## Results and Discussion

### Synthesis and Characterization of Poly(xylitol succinate)

**Figure 1:**
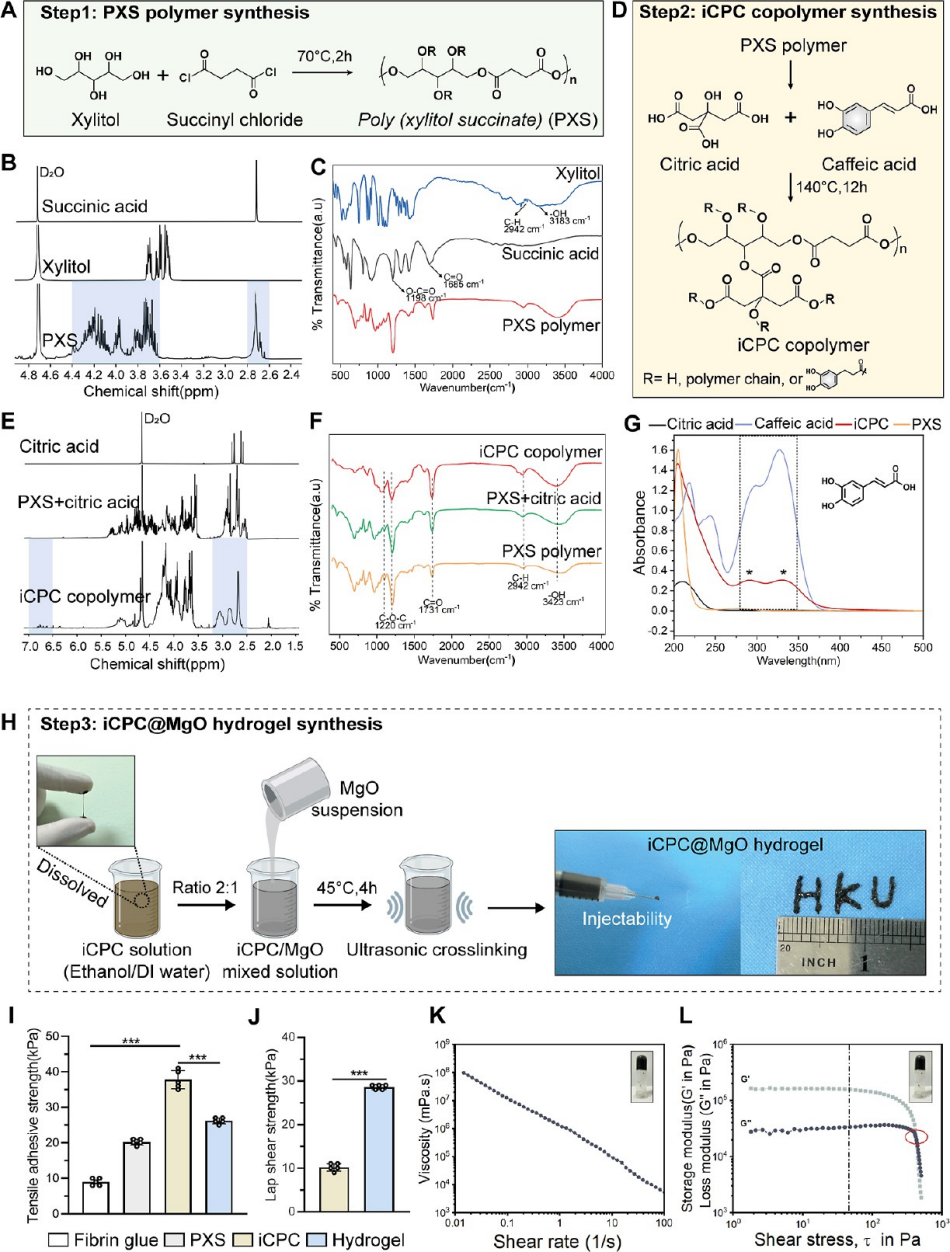
Synthesis and characterization of the polymers and injectable bioadhesive iCPC@MgO composite hydrogel. (A) Synthesis scheme of poly (xylitol succinate) (PXS) from xylitol and succinyl chloride. (B–C) NMR and FTIR analysis of PXS polymer. (D) Synthesis scheme of citric acid-poly(xylitol succinate)-caffeic acid (iCPC) copolymer undergoing the sequential esterification reaction. (E–G) NMR, FTIR, and UV–vis analysis of the iCPC copolymer, respectively. (H) Fabrication of the iCPC@MgO composite hydrogel through a cross-linking reaction involving MgO, using ultrasound mixing. (I) Tensile adhesive behavior of PXS, the iCPC copolymer, and the iCPC@MgO composite hydrogel compared to Fibrin glue. (J) Lap shear adhesive behavior of the iCPC copolymer and iCPC@MgO composite hydrogel. (K–L) Rheological testing of the iCPC@MgO composite hydrogel.

The iCPC copolymer was synthesized in two steps, as illustrated in Figure 1. Poly(xylitol succinate) (PXS) followed the same polymerization method as in previous literature (Figure 1A).^16,31^ The ^1^H NMR and FTIR spectra of PXS and two starting materials are provided in Figure 1B,C. The ^1^H NMR spectrum analysis of PXS showed the two obvious zones in Figure S1. Zone 1 represented multiple alkyl proton signals of the xylitol portion, divided into three main peak positions: the multiple chemical signals at 4.19 ppm (position a, Figure S1) were ascribed to two methylene signals forming the ester group in the polymer. The broad peak at 3.97 ppm was ascribed to the tertiary methyl proton signal connecting the hydroxyl groups on both sides (position b). The peak at 3.72 ppm (position c) was ascribed to the tertiary methyl proton signal connected by the middle hydroxyl group in xylitol. The main signals within the Zone 2 range were ascribed to the polymer’s two methylene-CH_2_ protons of succinic acid (Figure S1). The presence of multiple signals in this region confirmed the diversity of the polymer structure. The peaks of succinic acid found at 1685 and 1198 cm^–1^ could be attributed to C=O and O–C=O band stretching, and xylitol showed its characteristic OH stretching at 3183 cm^–1^. After polymerization, peaks were observed at 1220 cm^–1^, 2942 cm^–1^, and 3423 cm^–1^, respectively, which exhibited the characteristic bands of C–O–C, C–H, and O–H. The molecular weight of PXS was measured by gel permeation chromatography (GPC) as the *M*_n_ values are 687 kDa, and *M*_w_ is 697 kDa. These results are consistent with previous studies, indicating accurate synthesis of the polyester compound that corresponds well to its structure.^16,31^

### Synthesis and Characterization of the iCPC Copolymer

Citric acid-poly(xylitol succinate)-caffeic acid (iCPC) was prepared via an esterification reaction of PXS followed by dehydration condensation and chain extension to form a network structure. The schematic diagram and ^1^H NMR spectra of this compound are shown in Figures 1D and S2. From ^1^H NMR results (Figure 1E), the proton signals of caffeic acid involved in polymerization showed peaks at 5.80 to 6.80 ppm: the peaks at 6.82 and 5.87 ppm corresponded to the proton signals of the olefin, and multiple signals in the range of 6.37–6.70 ppm corresponded to the benzene ring protons of caffeic acid. The multiple signals around 5.00–5.30 ppm corresponded to the proton signals of the free hydroxyl groups and some methine groups of xylitol moieties in the polymer. The strong signals at 3.50–4.40 ppm corresponded to the proton of the methylene groups connected to various oxygen atoms from the xylitol moieties in the polymer, which confirmed that xylitol is the main constituent unit. The two main signals at 2.50–3.20 ppm corresponded to the methylene protons of the succinic acid moieties in the polymer, and the single peak at 2.69 ppm corresponded to the methylene protons of citric acid involved in the polymerization (Figure S2).

FTIR results showed that the O–H stretching band intensity increased at 3423 cm^–1^ for the iCPC copolymer in the spectra, indicating the increasing catechol group from caffeic acid (Figure 1F). The absorbance changes of iCPC at each wavelength of the UV–vis spectra are shown in Figure 1G; the two characteristic peaks from caffeic acid on the iCPC copolymer were observed around 280 and 330 nm; it also exhibited a marked absorption peak at 210 nm from the poly(xylitol succinate). The spectra and absorption band changes in the ^1^H NMR and FTIR data confirmed the successful introduction of caffeic acid in the iCPC composite polymer.

### Synthesis and Characterization of the iCPC@MgO Composite Hydrogel

The divalent transition metal ions have been proven to cross-link with 4 arms of polyethylene glycol-dopamine in viscoelastic gel networks.^32^ The cross-linking reaction mechanism and procedures are depicted in Figure 1H. The upturned trial indicated that the iCPC@MgO composite hydrogel was successfully fabricated through a MgO metal–ligand cross-link reaction via ultrasound mixing of the solution. During the cross-linking process, MgO and its release of Mg^2+^ might form covalent bonds with the catechol groups in the iCPC copolymer.^33^ Ultrasound vibration has also been proven to assist in forming hydrogels by inducing the cross-linking process.^34^ The injectable behavior of the hydrogel was visualized using a 1 mL syringe. The injectability of the hydrogel makes it a great candidate as a dental material due to the uneven shape of the tooth, periodontal defects, and ease of operation in the clinic.

The lyophilized hydrogel sample displayed a porous network structure (Figure S3). Considering the narrow space across the periodontal space, the swelling properties of the hydrogel are crucial. As confirmed by a quantitative survey, the maximum swelling ratio of this hydrogel reached about 167% of its weight, and the swelling plateaued from 12 h onward (Figure S3). The degradation assay also found that the hydrogel could rapidly degrade 24% within 2 days, and over half of the degradation rate was reached after 21 days (Figure S4A). This degradation rate implied that interrupted injections would be required for at least one month to achieve bone formation effects in the in vivo study.^35^

Although the tensile adhesive strength of the cross-linked hydrogel was not higher than that of the iCPC copolymer (Figure 1I), its lap shear adhesion behavior was reinforced compared to that of the iCPC copolymer (Figure 1J). Lap shear stress is a more relevant parameter for periodontal therapies, experienced by PDL cells during tooth movement, influencing cell behavior and osteogenesis.^36,37^ The high adhesiveness of the hydrogel is likely attributed to the iCPC composite polymer that was derived from the catechol groups provided by caffeic acid and the interaction of MgO with catechol groups or carboxyl groups of the iCPC copolymer to form the surface bonding or hydrogen bonding. The adhesive characteristic of the hydrogel helps optimize the antibacterial effect of medical applications by containing dynamic covalent bonds (ester bonds) or acidic carboxyl functional groups.^38^ The iCPC@MgO composite hydrogel in this study showed an enhancement of adhesion strength compared with the poly(ethylene imine)-based hydrogel, which has previously been reported for treating periodontitis.^39^ In Figure 1K,L, the rheological test showed that the storage modulus (*G*′) significantly increased from 1% to 2% in iCPC@MgO compared with the hydrogel presolution (Figures S4B and S5). This phenomenon confirmed that the chelation of ultrasound could act as an efficient cross-linking method to improve the mechanical properties of the hydrogel. The hydrogel could achieve a slow release of Mg^2+^ over 4 weeks in vitro (Figure S4C).

### Poly(xylitol succinate) Is a Competitive Antagonist of the TLR4-MD2 Complex

**Figure 2:**
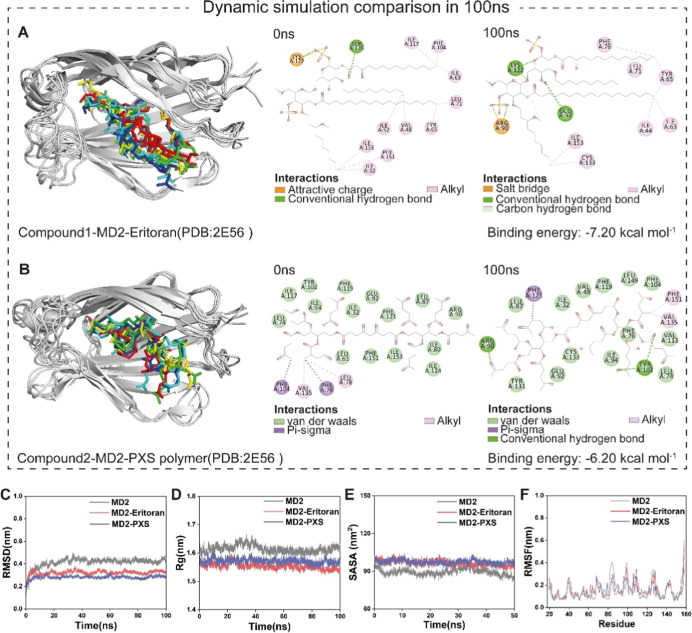
PXS polymer exhibits a binding affinity to MD2 protein comparable to Eritoran. (A,B) Representative interaction sites of MD2-PXS and MD2-Eritoran for molecular dynamics in 100 nm. Gray represents the MD2 protein. (C) Time series of the RMSD during 100 ns in groups of MD2, MD2-PXS, and MD2-Eritoran. (D) The *R*_g_ values for compactness of the MD2 protein with PXS, and Eritoran during 100 ns simulations. (E) SASA plot analysis corresponding to 100 ns simulations of MD2-PXS and the MD2-Eritoran complex. (F) The RMSF of MD2-PXS and MD2-Eritoran during 100 ns simulations. The *x*-axis represents the total number of residues. MD2, myeloid differentiation factor 2; RMSD, root-mean-square deviation; *R*_g_, radius of gyration; SASA, solvent-accessible surface area; RMSF, root-mean-square fluctuation.

We conducted a molecular docking simulation analysis to predict that the PXS polymer combats inflammation. We aimed to compare the interaction energy between the PXS polymer with Eritoran, a synthetic lipid A endotoxin antagonist that prevents LPS from binding to the cell surface TLR4-MD2 complex.^40^ Our study used Eritoran as a positive comparison group.^41^ MD2 is a protein that associates with the extracellular domain of Toll-like receptors 4 (TLR4), and it is thought to be the main component of the TLR4-MD2 complex that interacts with LPS to promote inflammation and stimulate the release of IL-6 by activating the NF-κB pathway.^42^ The dimer structure of PXS was used for docking with the MD2 protein (Figure S6). Through computational docking, a detailed understanding of the mechanism of PXS can be supported by strong evidence derived from the comparative analysis of binding sites, forces, and energies between PXS and established anti-inflammatory compounds with MD2.^43^ As shown in Figure 2A,B, the connection between MD2-PXS and MD2-Eritoran was driven by intermolecular interactions, including hydrogen bonds (H-bonds), π interactions, and the van der Waals force. There were no significant changes in the types of interaction forces between the ligand and the protein, indicating that the MD2-PXS complex exhibits high stability. Interestingly, we found that the PXS polymer has a similar bonding affinity to Eritoran, ranging from 6.20 to 7.20 kcal mol^–1^, which falls within the acceptable unsigned error for binding energies.^44^

We compared the protein interactions and stability of the PXS polymer to those of Eritoran through various analyses, including root-mean-square deviation (RMSD), radius of gyration (*R*_g_), solvent-accessible surface area (SASA), and root-mean-square fluctuation (RMSF). RMSD analysis helps us understand the movement of atoms in the protein domains during the simulation. Smaller deviations indicate a more stable protein structure.^45^ Based on the RMSD analysis in Figure 2C, MD2-PXS remained stable throughout the 100 ns simulation, while MD2 showed more fluctuations. We also looked at the *R*_g_ values. A high *R*_g_ value suggests instability. Interestingly, although there was no significant difference in the average *R*_g_ values between the PXS and Eritoran groups (1.57 ± 0.013 vs 1.56 ± 0.015 nm) (Figure 2D), both values were lower than MD2. The average SASA values for the MD2-PXS and MD2-Eritoran complexes during the 100 ns simulations were 99.64 and 99.35 nm^2^, respectively (Figure 2E). Data analysis indicated no significant difference in the SASA values between the two groups. RMSF, which measures the average deviation of protein residues over time from a reference position,^46^ indicated that MD2-Eritoran had more flexible residues at positions 100 to 120 compared to the MD2-PXS system, suggesting greater movement (Figure 2F). Based on these results, it can be concluded that MD2-PXS demonstrates protein interaction and stability similar to those of Eritoran.

**Figure 3:**
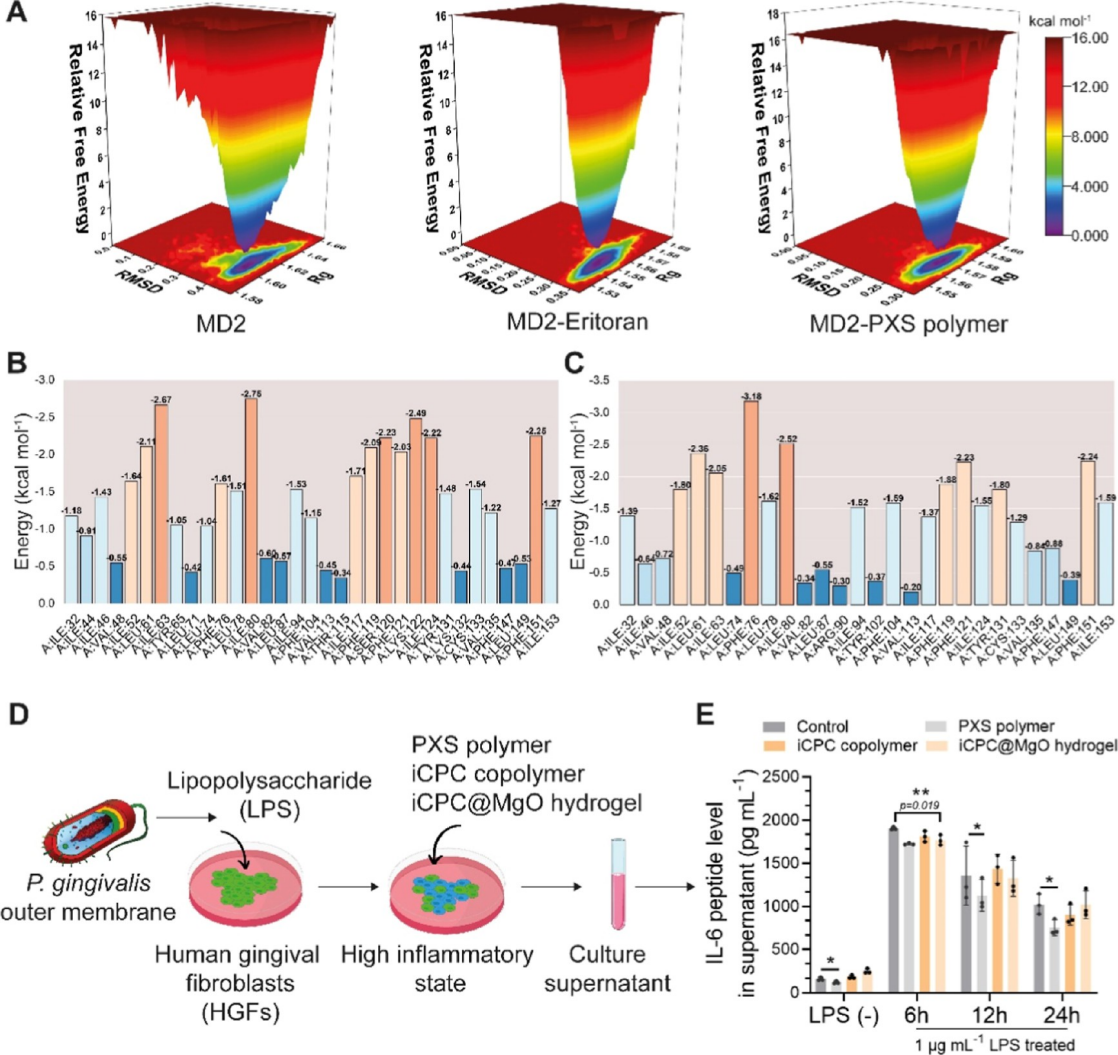
PXS polymer acts as an anti-inflammatory antagonist. (A) The FEL diagram involves the *R*_g_ and RMSD values for molecular dynamics in 100 nm. Free energy values are represented in kcal mol^–1^, and their colors are detailed in the color bar. (B,C) Binding energy contribution of the amino acid residues in MD2-PXS (left) and MD2-Eritoran (right) complex during molecular dynamics. (D) Schematic diagram of the inflammatory stimulation procedure by LPS-p.g for HGFs. (E) ELISA results for IL-6 after being treated with PXS polymer, iCPC copolymer, and iCPC@MgO hydrogels at 6 and 12 h (*n* = 3 biologically independent replicates). MD2, myeloid differentiation factor 2; FEL, free energy landscape.

In order to understand the distribution of binding energy in a protein molecular complex, 3D plots of the free energy landscape (FEL) were created by using two reaction coordinates: RMSD and *R*_g_. The lowest free energies are shown in blue, indicating more stability, while higher free energies are represented by other colors, indicating instability in the binding process.^47^ In FEL diagrams, strong and stable interactions can be observed in the MD2-Eritoran and MD2-PXS complex, shown by less green compared with MD2 (Figure 3A). The contribution of binding energy from the amino acid residues involved in the MD2-PXS and MD2-Eritoran complexes is illustrated in Figure 3B,C. The frequencies of amino acids in MD2-PXS are similar to those in MD2-Eritoran. The comparison results indicate that the PXS polymer has the potential for antagonistic ability against TLR4, similar to that of the Eritoran ligand.

### Poly(xylitol succinate) Suppresses IL-6 Release and Exerts Anti-inflammatory Effects by Inhibiting the NF-kappaB Pathway

Human gingival fibroblasts (HGFs) play an important role in the development and progression of periodontitis. The inflamed HGFs may recruit and activate inflammatory cells while maintaining persistent inflammation, leading to the degeneration of tooth-supporting structures.^48^ Therefore, we used HGFs to test the anti-inflammatory effects of the hydrogel (Figures 3D and S7). When HGFs were treated with the pro-inflammatory molecule lipopolysaccharide (LPS), they showed increased levels of IL-6. IL-6 has been well characterized as a major player in chronic periodontitis, and the expression of IL-6 is upregulated in the initiation phase of periodontitis.^49^ However, after being treated with the iCPC@MgO composite hydrogel, HGFs exhibited reduced IL-6 levels compared to the control group, and the PXS polymer group significantly inhibited IL-6 protein release at 6, 12, and 24 h (Figure 3E). Even the LPS-nontreated groups, when treated with the PXS polymer, showed inhibited IL-6 release, suggesting that the PXS polymer may be the primary functional component in controlling inflammation.

**Figure 4:**
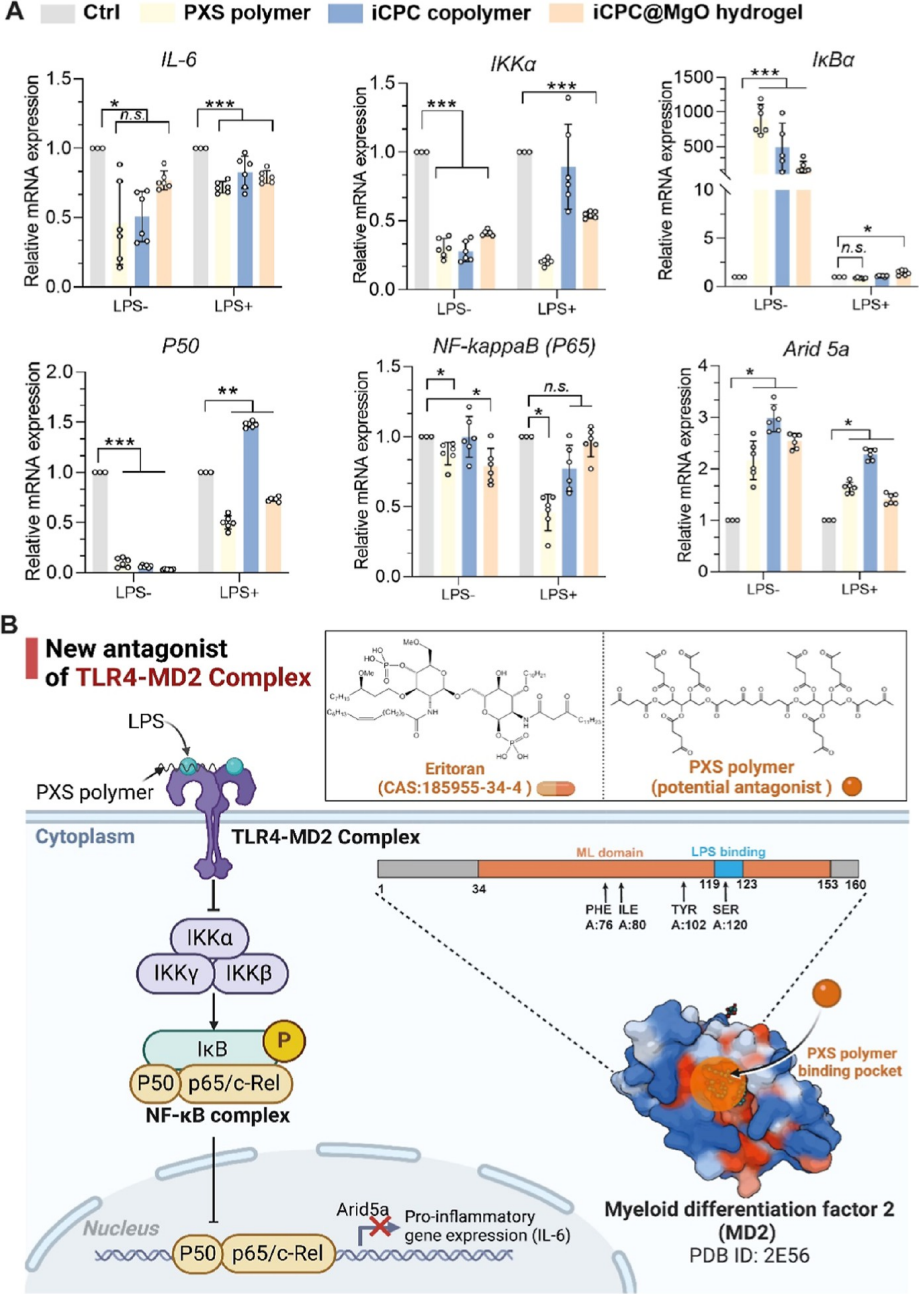
PXS polymer inhibits the IL-6 release via NF-κB pathway suppression. (A) qPCR results of mRNA expression levels of genes of the NF-κB pathway after stimulation by 1 μg mL^–1^ LPS and without LPS stimulation. (*n* = 6 biologically independent replicates). HGFs, human gingival fibroblasts. Data are mean ± s.d. Statistical significance was analyzed by one-way ANOVA (*p < 0.05, **p < 0.01, ***p < 0.001, ns represents nonsignificance). (B) Schematic diagram of the proposed anti-inflammatory mechanism of the PXS polymer. The PXS polymer competes to bind with the MD2 protein, thereby preventing the lipopolysaccharide from binding to the TLR4 receptor, ultimately inhibiting the NF-κB pathway. Specifically, the PXS polymer forms a chemical bond with the serine site of the MD2 protein at the binding pocket.

Additionally, we found that the PXS polymer produces anti-inflammatory effects by inhibiting the nuclear factor-κB (NF-κB) pathway. We assessed the mRNA expression levels of different genes responsible for the anti-inflammatory response in groups treated with LPS compared with those without treatment (Figure 4A) in hydrogel-treated groups. From the gene expression results of NF-κB inhibitor α (IκBα), p65 (RelA), p50 (NFκB1), IκB Kinase α (IKKα), and Arid 5a, it was clear that when pretreated with LPS, the level of IKKα was significantly reduced in PXS groups compared with the control (0.201 ± 0.025, *p* < 0.001), this is in line with the finding from previous research.^50^ This led to a decrease in P50, which, in turn, suppressed the NF-κB pathway (Figure 4A). Even in the absence of LPS stimulation, PXS has been shown to reduce the mRNA and protein expression of IL-6 (Figure 4A). However, what distinguishes it from the presence of LPS is the significant increase in IκBα levels in the PXS polymer group (896.47 ± 211.67, p < 0.001), which also has inhibitory effects on the NF-κB pathway (Figure 4A). As supported by previous reports, the activation of NF-κB not only relies on the inducible degradation of IκBα but also on a strong negative feedback loop of IκBα that can remove NFκB from the nucleus.^51−53^

Our findings indicate that the PXS molecule, which could be a TLR4-MD2 antagonist, has anti-inflammatory effects by inhibiting the NF-kappaB pathway and reducing the release of IL-6. The binding site of PXS with the amino acids of the MD2 protein is partially located within the structural domain, which increases the possibility of changes in the protein functionality (Figure 4B).

### iCPC@MgO Composite Hydrogel Displays Excellent Biocompatibility and Promotes Osteogenic Differentiation of hPDLSCs

**Figure 5:**
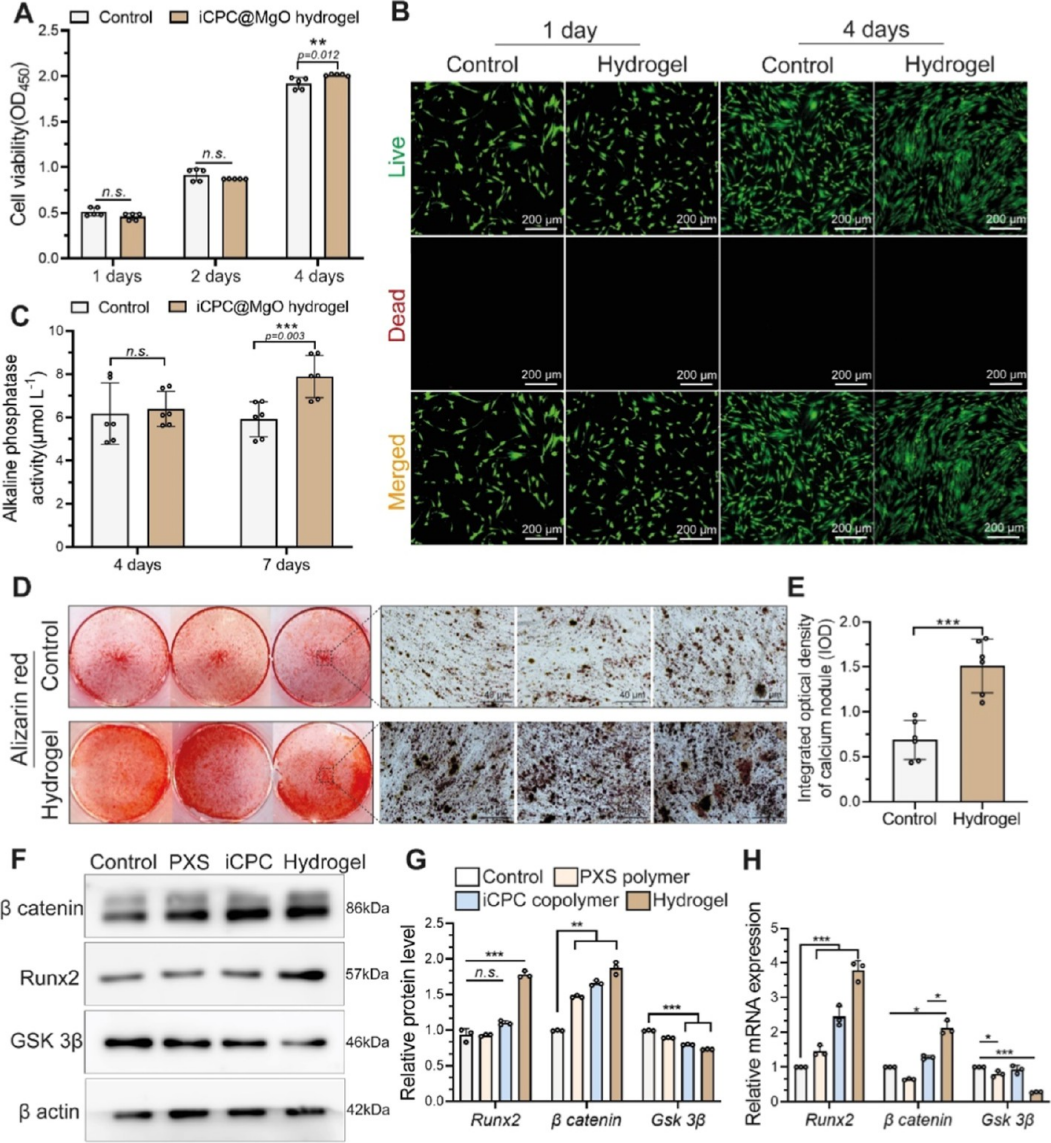
Biocompatibility of the iCPC@MgO composite hydrogel and its effects on hPDLSCs osteogenic differentiation. (A) CCK8 assay and (B) live/dead staining for hPDLSCs’ viability after culturing on the 4 mg mL^–1^ hydrogel in each well. (C) Alkaline phosphatase activity assay was used to evaluate the early osteogenic abilities after culturing on 4 mg mL^–1^ hydrogel at 4 and 7 days. (D) ARS at 21 days of culturing on the 4 mg mL^–1^ hydrogel. (E) Semiquantification of calcium nodules (*n* = 6 biologically independent replicates). (F,G) Western blot assay to detect the protein levels of the Wnt/β-catenin pathway at 21 days of culturing on the hydrogel. (H) Runx2, β catenin, and GSK 3β relative mRNA expression in hPDLSCs at 21 days of culturing on the hydrogel. Data are mean ± s.d. Statistical significance was analyzed by one-way ANOVA (*p < 0.05, **p < 0.01, ***p < 0.001, ns represents nonsignificance).

In addition to the adhesive properties and anti-inflammatory effects of an injectable hydrogel, two important factors for periodontal applications are cytocompatibility and osteogenicity. These factors play a therapeutic role in periodontitis by promoting the repair of the associated alveolar bone defects. Before further assessing the osteogenic ability of the hydrogel, the sterilized hydrogel was injected into the culture dishes to assess the biocompatibility on human periodontal ligament cells (hPDLSCs) by the CCK8 assay and live/dead staining. After 1 and 2 days of incubation, no adverse effects on cell viability were observed in the hydrogel group, while the cell proliferation rate was higher on day 4 compared with the control (Figure 5A). Similarly, live/dead staining also confirmed its excellent cytocompatibility (Figures 5B and S8). These biocompatible properties could be attributed to our naturally occurring starting materials: caffeic acid is naturally produced through the metabolism of vegetables or plants, and a five-carbon sugar alcohol, xylitol, is also used in the food industry.^54,55^ To assess the periodontal regeneration abilities, HGFs and hPDLSCs were used in scratch assays, and the results revealed that the migration rates of two periodontal cell types significantly increased after 12 and 24 h of coculture with the iCPC@MgO hydrogel compared to the control group (Figure S9). This finding suggests that iCPC@MgO enhances the migratory capacity of the periodontal cells.

When hPDLSCs were cultured on the hydrogel surface under an osteogenic induction medium, at 7 days, they displayed a significant increase in alkaline phosphatase levels, indicating an enhanced early osteogenic capacity (Figure 5C). The superior mineralization effects were also observed, as shown by significantly higher Alizarin red-stained (Figure 5D) calcium nodules in the hPDLSCs cultured on the iCPC@MgO composite hydrogel group than in the control group (Figure 5E). Accordingly, the expression of β catenin and Runx2 proteins was significantly increased in the iCPC@MgO composite hydrogel group, together with a significantly lower expression of GSK 3β ion channels when compared to the other three groups (Figure 5F,G). GSK 3β has been identified as a negative regulator of Wnt/β-catenin, which is known to be a key inducer of bone formation.^56^ The inhibition of GSK 3β results in β catenin accumulation in the cytoplasm and then promotes the expression of osteoblast-specific genes, including Runx2.^57^ Mg^2+^ has been proven to enhance the alveolar bone regeneration in vivo and in vitro by active β catenin levels and activation of Wnt/β-catenin target genes,^58^ which is consistent with our mRNA expression results in Figure 5H. Additionally, previous evidence indicated that activation of the canonical Wnt signaling pathway, induced by Mg^2+^ in the bone marrow space, prompts mesenchymal stem/stromal cells (MSCs) to differentiate into the osteoblast lineage.^59^ Moreover, the addition of citric acid to the iCPC copolymer allowed the hydrogel to form aqueous conditions, which could exert mild etchant effects on the surrounding alveolar bone to increase the spreading of preosteoblasts.^60^ Therefore, in addition to acting as a cross-linker in the hydrogel, incorporating MgO into the slightly acidic polymer may have synergistically improved its osteogenic properties.

### iCPC@MgO Composite Hydrogel Effectively Eliminates *Aggregatibacter actinomycetemcomitans* and *Porphyromonas gingivalis* by Stimulating Antibiotic Synthesis within Bacteria and Disrupting the Bacterial Cell Membrane

**Figure 6:**
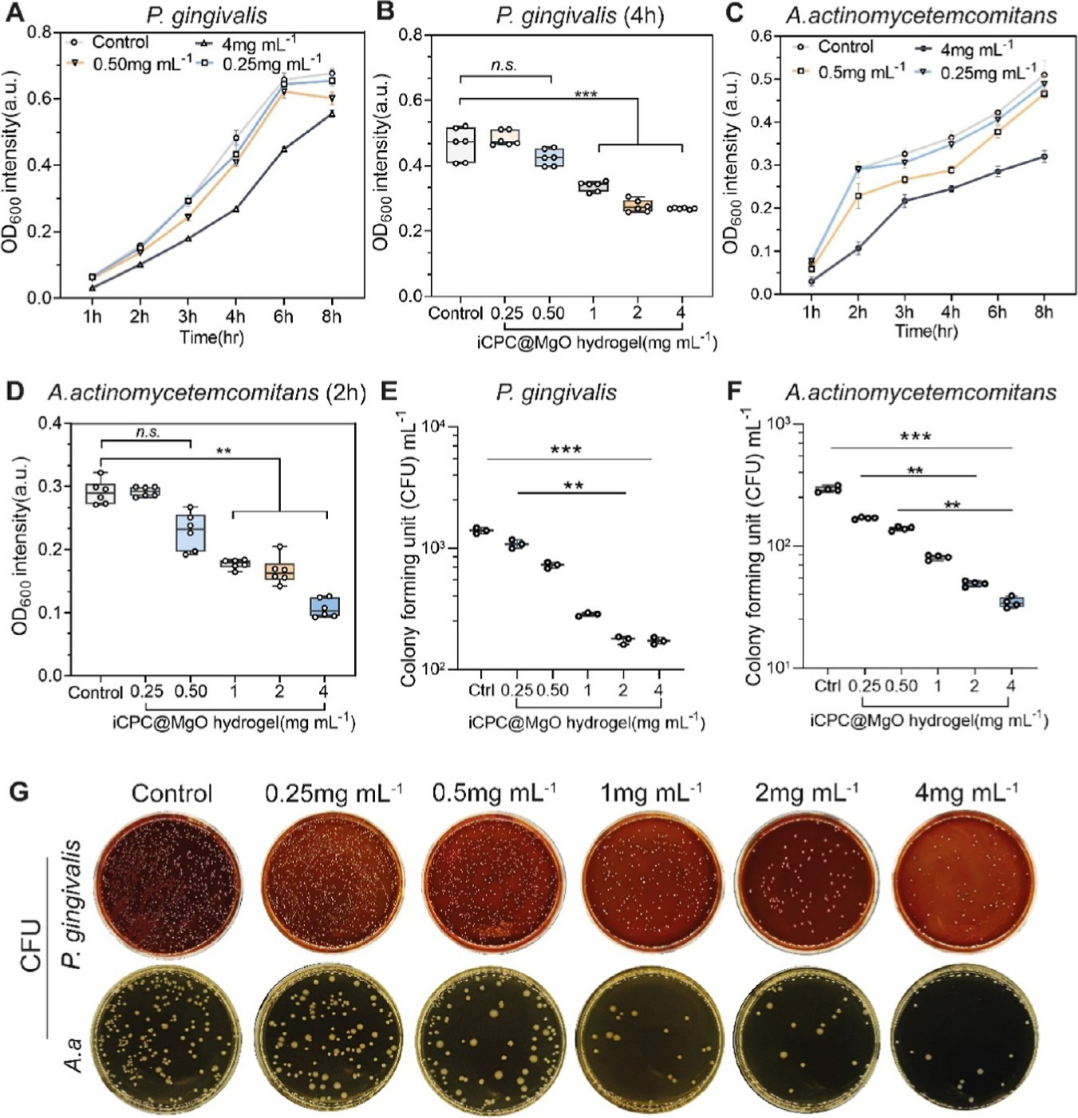
iCPC@MgO composite hydrogel effectively eliminates *A. actinomycetemcomitans*and *P. gingivalis*. (A) OD at 600 nm of *P.g* bacterial suspension after exposure to various hydrogel concentrations over 8 h. (B) OD at 600 nm of *P.g* bacterial suspension after exposure to various hydrogel concentrations at 4 h. (C) OD at 600 nm of *A.a* bacterial suspension after exposure to various hydrogel concentrations over 8 h. (D) OD at 600 nm of *A.a* bacterial suspension after exposure to various hydrogel concentrations at 4 h. (E–G) Enumeration of bacterial CFUs after exposure to different hydrogel concentrations at 24 h. CFUs, colony forming units. Data are mean ± s.d. Statistical significance was analyzed by one-way ANOVA (*p < 0.05, **p < 0.01, ***p < 0.001, ns represents nonsignificance).

**Figure 7:**
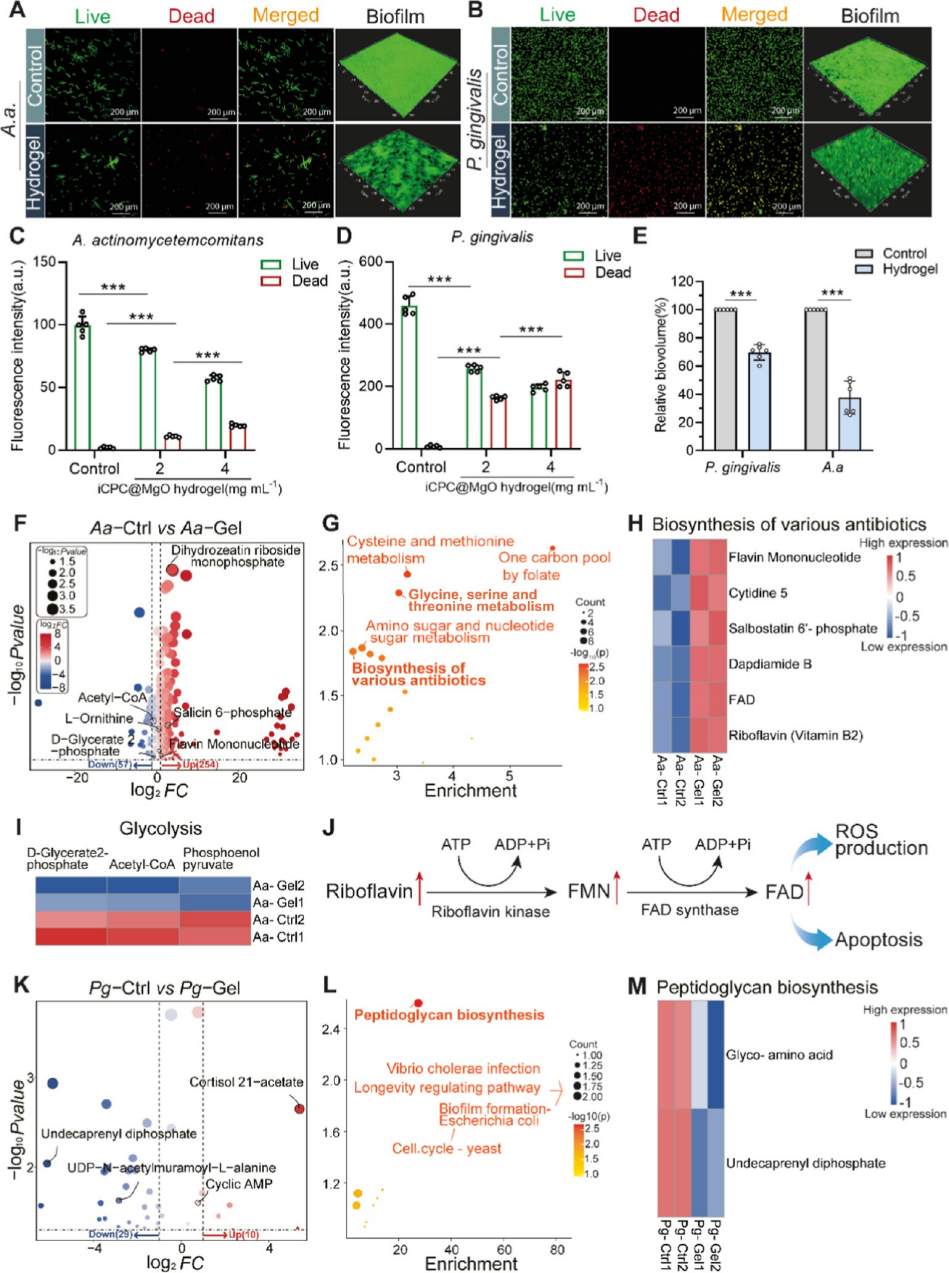
iCPC@MgO composite hydrogel exhibits bactericidal activity by enhancing antibiotic synthesis and damaging peptidoglycan synthesis. (A,B) Representative 2D and 3D live/dead-stained CLSM images of *A.a* and *P.g* after exposure to 4 mg mL^–1^ hydrogel. (C,D) Quantified fluorescence intensity of live/dead staining following treatment at 2 and 4 mg mL^–1^ hydrogel at 24 h. (E) Relative biovolume of *A.a* and *P.g* biofilms incubated, green-stained (live bacteria) after being treated with 4 mg mL^–1^ hydrogel at 7 days. (F) A volcano plot of differentially expressed metabolites in *A.a* bacteria following 4 mg mL^–1^ hydrogel treatment at 24 h. The control group was treated with PBS. (G) KEGG enrichment analysis showing the top 5 significantly enriched metabolic pathways for *A.a* bacteria. (H) Cluster map showing differential expression of metabolites involved in the enhancing antibiotic synthesis pathway. Red: high-expression; blue: down-expression. (I) Cluster map showing differential expression of metabolites with the downregulation glycolysis. (J) The increasing expression of FMN and FAD is synthesized from riboflavin in the 4 mg mL^–1^ hydrogel group compared with the control. The enhancement of the riboflavin biosynthesis pathway implies that it may be a crucial mechanism for the hydrogel to exhibit its antibacterial activity. (K) A volcano plot of differentially expressed metabolites in *P.g* bacteria following 4 mg mL^–1^ hydrogel treatment at 24 h. The control group was treated with PBS. (L) KEGG enrichment analysis in *P.g* bacteria. (M) Cluster map showing differential expression of metabolites involved in the downregulation peptidoglycan biosynthesis pathway. Red: high-regulation; blue: down-regulation. FMN, flavin mononucleotide; FAD, flavin adenine dinucleotide. Data are presented as mean ± s.d. Statistical significance was analyzed using one-way ANOVA (*p < 0.05, **p < 0.01, ***p < 0.001, ns indicates nonsignificance).

We chose *P. gingivalis* (P.g) and *A. actinomycetemcomitans* (A.a), the two predominant periodontal pathogenic bacteria, as the subjects for evaluating the antibacterial effectiveness of the hydrogel.^61^ At a concentration of 4 mg mL^–1^, the hydrogel inhibited the growth of P.g within 8 h, as compared to the control group (Figure 6A). The minimal inhibitory concentration (MIC) of the iCPC@MgO composite hydrogel against P.g was 1000 μg mL^–1^ at 4 h (Figure 6B). Similarly, the 4 mg mL^–1^ hydrogel exhibited the inhibition of bacterial growth within 8 h for A.a (Figure 6C). The minimal inhibitory concentration (MIC) of the hydrogel against A.a was 500 μg mL^–1^ at 2 h (Figure 6D). Subsequent enumeration of colony-forming units (CFUs) also indicated that the hydrogel exhibits an increased antibacterial efficiency at higher concentrations, as evidenced by progressively decreasing CFU numbers (Figure 6E–G). In addition, live/dead staining was performed to distinguish dead from live bacteria and evaluate the bactericidal properties of the iCPC@MgO composite hydrogel (Figure 7). As depicted in Figure 7A to D, the population of red fluorescent-stained bacteria increases, indicating a significant reduction in bacterial count after coculturing with the hydrogel. Due to the biofilm-modulated development of periodontitis, destroying bacterial biofilms is the initial step toward effectively controlling the inflammatory reaction.^62^ The relative biovolume of the *P.g* and *A.a* biofilms treated with 4 mg mL^–1^ hydrogel at 7 days is significantly lower than that of the control group (Figure 7E). This suggests that the iCPC@MgO composite hydrogel effectively disrupts the biofilm formation.

Metabolomics analysis was conducted to understand how the hydrogel affects biofilm formation and bacterial activities. The analysis revealed differences in several metabolites between the hydrogel-treated and the control groups, providing insights into the mechanisms behind the antibacterial properties of the iCPC@MgO composite hydrogels. In groups treated with 4 mg mL^–1^ hydrogel, there was a significant increase in metabolites related to antibiotic production in *A.a* bacteria, including *N*-formylmethionine (fMet), l-ornithine, and flavin mononucleotide (FMN) (Figure 7F). The KEGG enrichment analysis revealed the top 5 differential expression pathways, among which the increased antibiotic synthesis provided an antibacterial mechanism for this hydrogel (Figure 7G,H). At the same time, metabolites associated with decreased glycolysis, such as *d*-glycerate 2-phosphate, were observed (Figure 7I). The increasing production of antibiotics could potentially improve antimicrobial effectiveness.^63^ For example, FMN is a precursor for synthesizing a class of antibiotics called aminoglycosides, which allows the antibiotics to bind to the ribosomes in bacteria and interfere with protein synthesis, ultimately leading to bacterial cell death.^64^ The specific metabolite concentration analysis showed that the increasing release of FAD is mainly due to upregulated riboflavin (Figure 7J). When glycolysis is decreased in bacteria, several consequences can occur, such as reduced energy production, impaired bacterial growth, proliferation, or altered metabolic flux.^65^ Our research shows that different types of bacteria respond differently to the same hydrogel. In particular, we noticed significant effects on the production of peptidoglycan and biofilm in *P.g* bacteria when they were treated with iCPC@MgO (Figure 7K,L). This suggests that iCPC@MgO has antibacterial properties by disrupting the bacteria’s cell membrane (Figure 7M). Additionally, we visualized the cell membrane disruption using TEM electron microscopy, as depicted in Figure S10. Decreased peptidoglycan synthesis can lead to a weakened cell wall, rendering the bacteria more susceptible to mechanical stress and environmental pressures.^66^ The results indicate that the iCPC@MgO composite hydrogel not only meets the performance requirements of adhesion and injectability but also successfully integrates various functional benefits as a whole.

Three raw materials with reported antimicrobial properties were used to create the iCPC@MgO composite hydrogel. Xylitol has been reported to contribute to reducing the accumulation of dental plaque by disrupting the energy cycle of bacteria.^67^ Similarly, caffeic acid has been suggested to exert antibacterial effects by interacting with its polyphenolic structures, leading to bacterial membrane damage.^68^ MgO nanoparticles exhibited antibacterial activity by inhibiting colony formation, which is attributed to the activation of oxidative stress in bacteria.^69^

### Effects of the iCPC@MgO Composite Hydrogel on Periodontitis Therapy In Vivo

**Figure 8:**
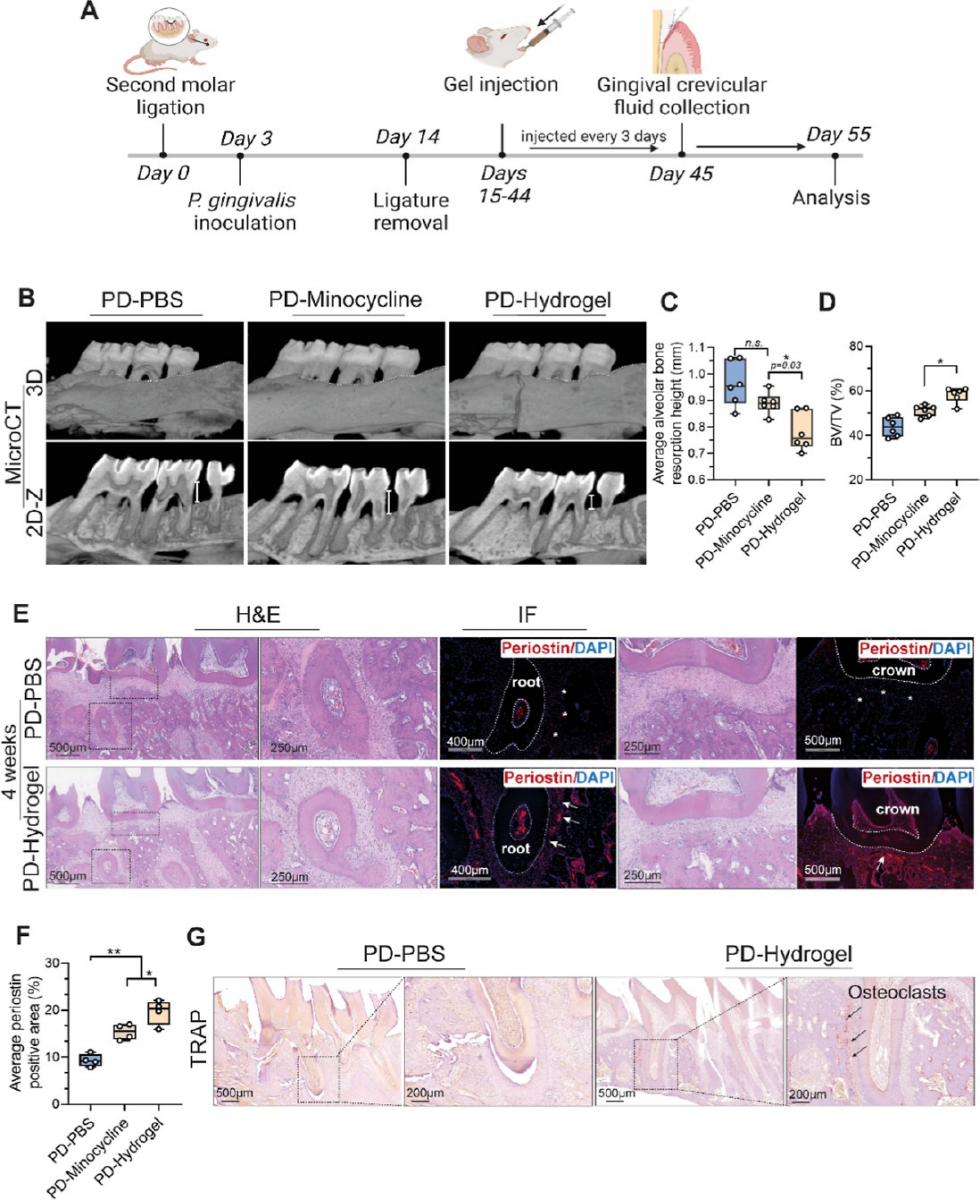
Evaluating in vivo osteogenic effects of the iCPC@MgO composite hydrogel in a rat periodontitis model. (A) Schematic illustration of the PD treatment schedule. The second molar was ligated for PD model creation. (B) Micro CT images of the radiographic bone loss for groups treated with PBS, minocycline hydrochloride (positive control group), and the iCPC@MgO composite hydrogel at 4 weeks (*n* = 5 biologically independent replicates). (C) The average alveolar bone resorption height (mm) in each group. (D) The BV/TV (%) using micro CT scanning in the surrounding alveolar bone of the second molar. (E) H&E-stained and immunofluorescent-periostin-stained sections of hydrogel and PBS-treated groups. (F) Quantitative results of periostin-positive area. (G) TRAP staining images of periodontium histological sections in different groups after 4 weeks. The minocycline hydrochloride group is included in the Appendix. PD, periodontal disease; BV/TV, bone volume/total volume of bone; IF, immunofluorescent. Data are presented as mean ± s.d. Statistical significance was analyzed using one-way ANOVA (*p < 0.05, **p < 0.01, ***p < 0.001, ns indicates nonsignificance).

To assess the hydrogel’s therapeutic efficacy in treating periodontitis in vivo, we first established and characterized the model of localized periodontitis via bilaterally ligating the maxillary second molar for 2 weeks (Figure 8A).^70^ Periodontitis parameters were measured in response to periodontal soft and hard tissue destruction, including the probing depth and gingival bleeding. The periodontitis model criteria demonstrated an increase in probing depth as the main symptom, apparent bone resorption, and bleeding upon gingival probing as a sign of gingival inflammation (Figure S11).

The micro-CT scan results revealed that the distance between the alveolar bone crest (ABC) and the cementoenamel junction (CEJ) was significantly lower in the teeth treated with the iCPC@MgO composite hydrogel (0.78 ± 0.071 mm compared to 0.96 ± 0.084 mm, *p* < 0.01) than in the antibiotic-exposed positive control group (minocycline) over the course of one month (Figure 8B,C). Accordingly, the bone volume/total volume of bone (BV/TV) increased by 57.5% in the hydrogel group (Figure 8D). When the sections were stained for periostin, the fluorescence intensity was twice as high in the alveolar bone crest areas of the hydrogel-treated group as in the control group (Figure 8E,F), indicating higher osteogenic activity. Furthermore, interestingly, TRAP staining detected an increased number of osteoclasts at the alveolar bone margins in the hydrogel-treated group compared with the control group (Figure 8G). It has been shown that in the rat periodontitis model, the number of osteoclasts increases up to 2 weeks after ligation and then gradually decreases.^70^ The hydrogel-treated group showed increased osteoclastic activity after one month of ligation, suggesting active bone formation and remodeling. This aligns with recent research indicating the crucial role of osteoclastic activity in osteoblastic bone formation.^71^ Taken together, our results showed that the iCPC@MgO composite hydrogel effectively alleviates alveolar bone loss in vivo.

**Figure 9:**
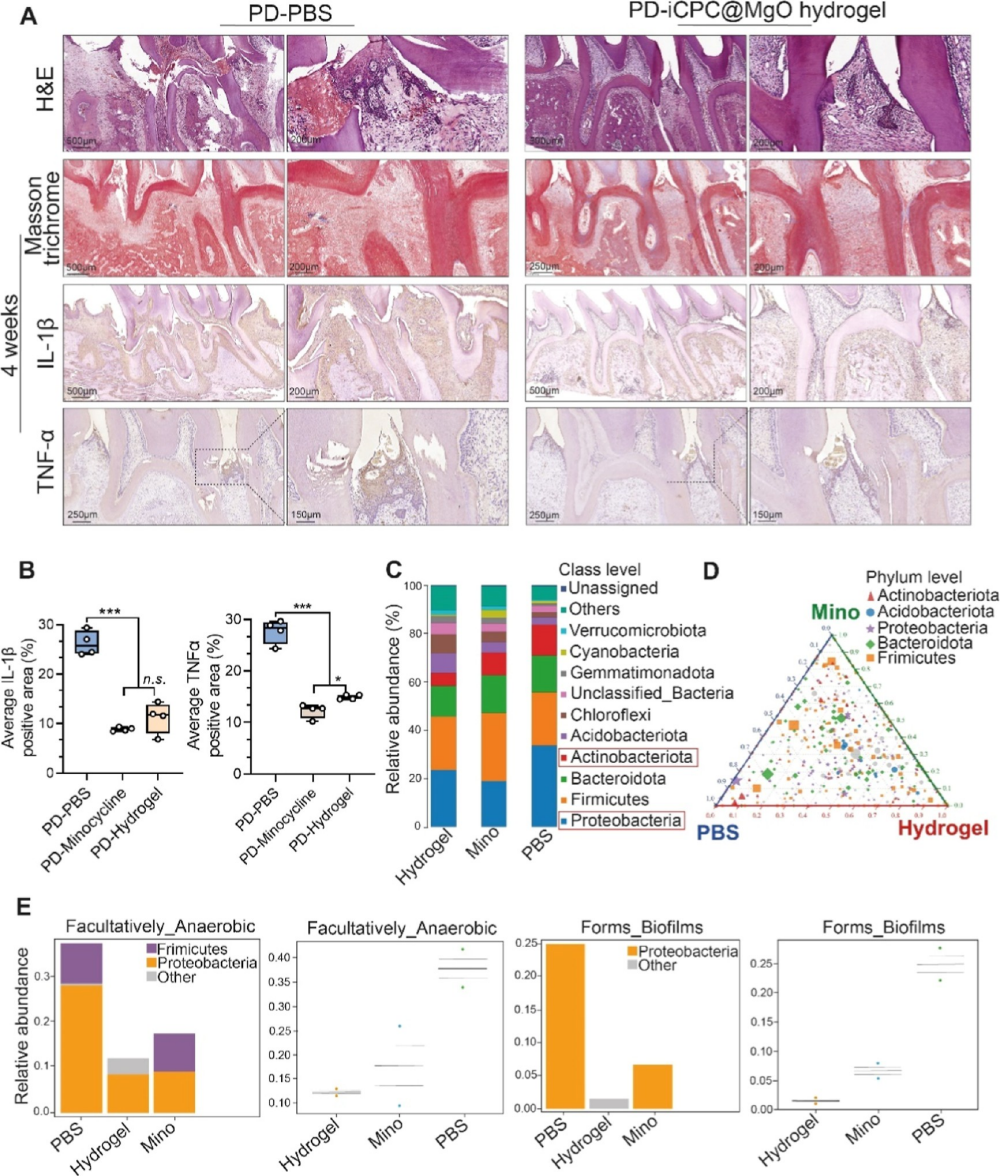
Evaluating in vivo anti-inflammatory and antibacterial effects of the iCPC@MgO composite hydrogel in the rat periodontitis model. (A,B) Histological evaluation of periodontal sections in PBS and hydrogel-treated groups (H&E staining, Masson trichrome staining, immunohistochemistry for IL-1β and TNFα, and quantification). (C–E) Microbiome sequencing results of gingival crevicular fluid collected at day 45 post-treatment. (C) Relevant abundant bacterial communities from the collected gingival crevicular fluid at the class level. (D) Ternary distribution of phylum classification. (E) Predicted phenotypes from different bacterial communities (*n* = 2). The minocycline hydrochloride group is included in the Appendix. PD, periodontal disease. H&E, hematoxylin and eosin. Data are presented as mean ± s.d. Statistical significance was analyzed using one-way ANOVA (*p < 0.05, **p < 0.01, ***p < 0.001, ns indicates nonsignificance).

Moreover, the study assessed the histology of periodontal tissues using hematoxylin–eosin and Masson’s trichrome staining, as well as the accumulation of pro-inflammatory factors through immunohistochemical (IHC) staining. H&E staining showed that the hydrogel group improved attachment of the gingival epithelium on the enamel surface and reduced inflammatory cell infiltration compared to the control group (Figure 9A). Masson’s trichrome staining showed well-organized collagen bundles in the periodontal ligament space surrounding the alveolar bone in the hydrogel-treated group. Furthermore, the IHC staining showed a significant reduction of pro-inflammatory factors, IL-1β and TNFα levels, in the periodontal tissues treated by the hydrogel (Figure 9A,B).

The gingival crevicular fluid from the gingival crevices (50 μL) of treated teeth was collected with absorbent paper on day 45 (Figure 8A). Using microbiome sequencing technology (16S) of the V3–V4 region, we evaluated whether the iCPC@MgO composite hydrogel affects the spatial structure of the subgingival microbiota. As shown in Figure 9C, the control group exhibited two dominant bacterial communities of periodontitis at the class level in gingival fluid: Actinobacteria and Proteobacteria, which were inhibited by the iCPC@MgO composite hydrogel. Ternary abundance analysis indicated that our hydrogel has no negative impact on the richness of subgingival bacterial communities (Figure 9D), which means it can preserve the balance of subgingival microbiota. Compared with the control and minocycline hydrochloride groups, these results showed a relative decrease in the abundance of Bacteroides, Firmicutes, and Proteobacteria, suggesting that our hydrogel reduced the number of periodontal pathogenic bacteria at the phylum level in vivo (Figure 9E). The hydrogel injection did not induce systemic toxicity in rats, as evidenced by histological staining (Figure S12), likely due to the hydrogel’s composition made from natural materials.

Taken together, the iCPC@MgO composite hydrogel shows great promise in periodontal therapy due to its natural and gentle antibacterial effect, ability to reduce surrounding inflammation, and promotion of bone tissue growth without causing dysbacteriosis.

## Conclusions

In summary, we developed an injectable multifunctional bioadhesive hydrogel system to address the inflammatory challenges in periodontitis directly. The synthesized poly(xylitol succinate) (PXS) polymer demonstrated notable TLR4-MD2 antagonism, surpassing the NF-kappaB pathway in anti-inflammatory efficacy and marking a significant advancement in inflammation control without additional drugs. The hydrogel also exhibited superior antibacterial and osteogenic properties both in vitro and in vivo studies, laying a strong foundation for future clinical periodontal regeneration. The natural components, PXS and iCPC polymers, may have a broadened range of applications in other fields. Enhancing its mechanical properties is important to expand the range of applications for the iCPC@MgO composite hydrogel. Additionally, addressing the limitations posed by the color of the hydrogel and optimizing the reaction process to enhance the grafting efficiency of caffeic acid will broaden its applicability in other oral applications.

## Methods

### Materials

Xylitol, succinate, citric acid, and caffeic acid were purchased from Dieckmann Chemical Co. Ltd., Hong Kong. Magnesium oxide, Alizarin red staining (ARS), alkaline phosphatase assay kit (ALP), LPS-p.g, 4′,6-diamidine-2′-phenylindole dihydrochloride (DAPI), Wiegert’s iron hematoxylin kit, phosphomolybdic acid solution, and Biebrich scarlet-acid fuchsin kit were the products of Sigma-Aldrich Co. Ltd., Germany. Human periodontal ligament cells (hPDLSCs) and human gingival fibroblasts (HGFs) were commercially obtained from the Elabscience Biotechnology Co. Ltd., Wuhan, China. Dulbecco’s modified eagle medium (DMEM), α-minimum essential medium (MEMα), brain heart infusion (BHI), fetal bovine serum (FBS), LIVE/DEAD cell imaging kit, LIVE/DEAD BacLight, pierce BCA protein assay kit (BCA), enhanced chemiluminescence (ECL) substrate, aniline blue, and avidin–biotin complex (ABC) kit were purchased from Thermofisher Scientific Inc., USA. Dexamethasone, ascorbic acid, β-glycerophosphate, and fibroblast growth supplement were purchased from Solarbio Science & Technology Co. Ltd., Beijing, China. The CCK8 kit was purchased from Beyotime Biotechnology Inc., Beijing, China. The RNA extraction kit, cDNA synthesis kit, and multiplex PCR kit were purchased from Qiagen, The Netherlands, and the Human IL-6 enzyme-linked immunosorbent assay (ELISA) kit was purchased from RayBiotech, Inc., USA. The primary antibodies for β catenin, GSK 3β, and Runx2 were obtained from Signalway Antibody Co. Ltd., US. IL-1β, TNF-α, and periostin primary antibodies were purchased from Proteintech, Inc., Wuhan, China. The tartrate-resistant acid phosphatase antibody (TRAP), IgG H&L secondary antibody (Alexa Fluor 647), IgG H&L (HRP) secondary antibodies, and 3,3′-diaminobenzidine (DAB) substrate kit were purchased from Abcam Co. Ltd., UK. Absorbent paper point (25#) was obtained from SHENGTENG Co. Ltd., China.

### Synthesis of Poly(xylitol succinate)

The synthesis of the PXS conjugate was referred to in the literature procedures with further modifications.^16,31^ The mixture of xylitol (0.45 g, 3 mmol, 1 equiv) and thionyl chloride (0.95 g, 8 mmol, 2.67 equiv) was heated at 70 °C for 1 h, and then succinic acid (0.35 g, 3 mmol, 1 equiv) was added to the reaction mixture. After stirring at 70 °C for 2 h and recovering to room temperature, the mixture was neutralized by 1 M NH_4_HCO_3_, redissolved in DI water, and extracted with dichloromethane, respectively. The solution was passed through a plug of silica gel, and afterward, the solvent was evaporated to get a transparent viscous product of PXS. The molecular weight of PXS was measured using gel permeation chromatography (GPC).

### Synthesis of the iCPC (Citric Acid–PXS–Caffeic Acid) Copolymer

PXS (4 g, 2 mmol, 1 equiv) and citric acid (0.65 g, 1 mmol, 0.5 equiv) were placed in a double-necked flask, and the reaction mixture was stirred and heated at 160 °C for 1 h under nitrogen protection. Then caffeic acid (0.54 g, 0.3 mmol, 6.67 equiv) was added to the mixture, and the temperature was reduced to 140 °C overnight. The produced prepolymer was purified by dialysis using the dialysis membrane (MWCO 500–1000 Da) in DI water for 7 days and, subsequently, freeze-dried before use. The raw materials and iCPC copolymers were dissolved in DI water to perform UV–vis absorption spectroscopy at a wavelength of 200–500 nm to get the conjugation efficiency of the iCPC copolymer.

### Preparation of iCPC@MgO Composite Hydrogels

The iCPC copolymer was initially dissolved in a mixing solution comprising ethanol and deionized (DI) water in a 4:1 ratio, achieving a 40 wt % polymer solution. Then, 20 wt % MgO was added to DI water, and the mixture was quickly shaken by hand. The iCPC@MgO composite hydrogel was prepared by combining the iCPC copolymer solution and the MgO dispersion in a volume ratio of 2:1.^33^ The prehydrogel solution was sonicated for 4 h to allow cross-linking at an average temperature of ∼45 °C. After freeze-drying the hydrogel, Fourier-transform infrared spectroscopy (FTIR) was carried out to confirm the chemical compositions of raw materials, iCPC copolymer, and iCPC@MgO composite hydrogel. The ^1^H NMR (400 MHz) spectra were recorded in D_2_O for PXS, caffeic acid, citric acid, PXS + citric acid, iCPC, and the iCPC@MgO composite hydrogel.

### iCPC@MgO Composite Hydrogel Validation

The validation of the iCPC@MgO hydrogel involved several assessments. The swelling ratio was determined by immersing dried samples in water and calculating the weight change. Degradation was evaluated by measuring mass loss in PBS over 21 days. The adhesive strength was tested on porcine skin under saliva conditions, and rheological properties were analyzed by using a dynamic rheometer with a parallel plate configuration. Detailed methodologies are provided in the Supporting Information.

### Computational Molecular Docking with the MD2 Complex

Computational molecular docking was utilized to evaluate the binding of PXS polymers to the MD2 protein by comparing it with the TLR4 antagonist Eritoran. The TLR4-MD2 structure was prepared by using PyMOL and Auto Dock Tools, followed by docking calculations with AutoDock Vina, achieving methodological validation with an RMSD of less than 2 Å. A 100 ns molecular dynamics simulation was conducted by using Gromacs to analyze protein–ligand interactions, incorporating energy minimization and equilibration steps. The simulations assessed various parameters, including RMSD, hydrogen bonds, and binding free energy, to evaluate the stability and interaction strength. Detailed methodologies are provided in the Supporting Information.

### Anti-inflammatory Property Evaluations

To simulate an inflammatory condition, human gingival fibroblasts (HGFs) were cultured in DMEM with 10% FBS and then treated with 1 μg mL^–1^ lipopolysaccharide (LPS) for 6, 12, and 24 h. To assess the anti-inflammatory effects of the polymers and hydrogel, HGFs were pretreated with LPS, followed by treatment with PXS polymer, iCPC copolymer, or iCPC@MgO hydrogel for 6, 12, and 24 h. Interleukin-6 (IL-6) levels in the culture media were quantified using ELISA. Additionally, total RNA was extracted for qRT-PCR to evaluate the expression of NF-κB pathway genes after 24 h. Detailed methodologies are provided in the Supporting Information

### Cytocompatibility Assay

Cell proliferation of hydrogels was assessed using CCK-8 and live/dead staining assays. hPDLSCs and HGFs were cultured in supplemented α-MEM and DMEM and then seeded onto the iCPC@MgO composite hydrogel in 96-well plates at a density of 3 × 10^4^ cells/well. After 1, 2, and 4 days, cell viability was measured via the CCK-8 assay with OD at 450 nm. For live/dead staining, cells were imaged using confocal microscopy with Calcein AM/iodide staining to visualize viability. Fluorescence intensities were quantified with ImageJ software. The scratch assay was conducted to assess the migration ability of hPDLSCs and HGFs. A scratch was created using a sterile pipet tip, followed by washing PBS to remove detached cells. Cells were then cocultured with the iCPC@MgO hydrogel, scratch area images were captured at 0, 12, and 24 h using an optical microscope, and the migration rate was quantified by measuring the gap area with ImageJ software.

### Alkaline Phosphatase Activity Assay

hPDLSCs were induced in vitro using MEMα medium supplemented with FBS, dexamethasone, ascorbic acid, and β-glycerophosphate. For ALP activity, the iCPC@MgO hydrogel was coated in a 12-well plate, solidified at 37 °C, and seeded with 10^4^ hPDLSCs per well. After 4 and 7 days in the osteogenic medium, the culture supernatant was collected. ALP activity was measured by transferring 50 μL of the medium into a 96-well plate, adding 150 μL of reagent, and reading the OD at 405 nm after 10 min.

### ARS Assay

For the ARS assay, hPDLSCs were seeded on an iCPC@MgO hydrogel in a 12-well plate and cultured for 21 days with medium changes every 3 days. After fixation with 4% PFA, cells were stained with Alizarin red for 30 min. Calcium nodules were quantified using ImageJ, and five different views were analyzed per well.

### Western Blotting and qRT-PCR Assay for the Wnt/β-Catenin Pathway

To evaluate the osteogenic effects of polymers and hydrogels, hPDLSCs were divided into four groups: the control group, PXS polymer, iCPC copolymer, and iCPC@MgO hydrogel. Cells were cultured in an osteogenic medium for 21 days before protein extraction using a RIPA buffer. Western blotting assessed Wnt/β-catenin pathway proteins (β-catenin, GSK 3β, and Runx2) on PVDF membranes. Additionally, total RNA was extracted for qRT-PCR to quantify gene expression following standardized protocols. Detailed information about antibodies and primers is provided in Tables S1 and S2.

### Antibacterial Properties of the iCPC@MgO Hydrogel In Vitro

The antibacterial efficacy of the hydrogel was evaluated against *P. gingivalis* and *A. actinomycetemcomitans* by using various assays. Hydrogel was diluted to concentrations of 4, 2, 1, 0.5, and 0.25 μg mL^–1^ for testing. Optical density (OD_600_) measurements assessed bacterial growth over 8 h. Colony-forming unit (CFU) counts were used to analyze bacterial viability after hydrogel treatment. Live/dead staining with SYTO 9 and propidium iodide evaluated bactericidal effects, while biofilm inhibition assays determined hydrogel effectiveness against biofilm formation over 7 days, with fluorescence imaging and COMSTAT 2 for analysis. Transmission electron microscopy (TEM) was used to examine microstructural changes in *P. gingivalis* after treatment with 4 mg mL^–1^ hydrogel. Bacterial suspensions were prepared, fixed, dehydrated, and observed under the microscope. Detailed methodologies are provided in the Supporting Information.

### Bacterial Metabolomics

To investigate the antibacterial mechanism of the iCPC@MgO hydrogel, bacterial metabolomics were performed on *P. gingivalis* and *A. actinomycetemcomitans*. The bacteria were incubated with the hydrogel, harvested, and prepared for analysis. Metabolites were extracted and analyzed by using liquid chromatography–mass spectrometry (LC–MS). Differential metabolites were identified, and pathway enrichment analysis was conducted by using KEGG to assess the biological pathways affected by hydrogel treatment.

### Periodontitis Model Establishment

All animal procedures were approved by Lanzhou University’s ethics guidelines (LZUKQ-2023-055). Eighteen male Sprague–Dawley rats (8 weeks old) were used to establish a bilateral molar periodontitis model using silk ligatures and *P. gingivalis* inoculation. Rats were divided into three treatment groups: PBS, minocycline gel, and iCPC@MgO hydrogel, with treatments administered every 3 days for 4 weeks. After 4 weeks, the rats were euthanized and maxillary bones were collected for further assays. Detailed methodologies are provided in the Supporting Information.

### Osteogenic Evaluation In Vivo

Micro-CT scans of the alveolar bone used Skyscan 1176 to assess bone volume fraction (BV/TV) around the second molar. The histological analysis involved hematoxylin and eosin staining (H&E), and immunofluorescence (IF) was performed for osteogenic properties. Bone sections were decalcified in 10% EDTA, permeated, and incubated with primary antibodies for periostin, followed by DAPI staining and visualization under a fluorescence microscope. TRAP staining identified osteoclasts, with sections incubated with TRAP antibodies and stained to visualize osteoclast presence in the alveolar bone.

### Anti-inflammatory Evaluations In Vivo

Masson’s trichrome staining: tissue sections were deparaffinized, rehydrated, and stained with Weigert’s iron hematoxylin for 10 min at room temperature. After washing, sections were stained with Biebrich scarlet acid fuchsin for 10 min, followed by differentiation in the phosphomolybdic acid solution for 15 min and staining with aniline blue for 10 min. The staining results were observed using an Olympus optical microscope (BX53, Japan).

### Immunohistochemistry

Immunohistochemistry (IHC) was conducted to detect the IL-1β and TNF-α levels in tissue sections. After deparaffinization and rehydration, the sections were blocked with 5% BSA. Then, the sections were incubated overnight at 4 °C with primary antibodies against IL-1β and TNF-α. Following three PBS washings, sections were treated with avidin–biotin complex (ABC) staining. Visualization was achieved using a DAB substrate kit; the images were observed using an Olympus BX53 optical microscope with mean positive expression areas quantified by ImageJ software.

### Microflora Difference in Gingival Crevicular Fluid

To assess the effect of the iCPC@MgO hydrogel on gingival microbiota, 16S rRNA sequencing was performed on samples collected from rat gingival crevicular fluid over 4 weeks. DNA was extracted, the V3–V4 region was amplified, and high-throughput sequencing data were generated. Bioinformatics tools were then used for analysis, including assessing microbial diversity and identifying significant variations in community composition.

### Statistical Analysis

All data were processed in SPSS (IBM SPSS Statistics 27.0.1). Each assay was conducted at least three times to ensure the accuracy of the results under identical conditions. Results were reported as the mean ± standard deviation (SD) and the percentage (%) as presented in the figures. The normality of the data distribution was assessed by using the Shapiro–Wilk test before performing statistical analyses. One-way ANOVA and Kruskal–Wallis statistical tests were used to evaluate group differences. *, **, ***, and **** denote a significant difference between groups of *P* < 0.05, *P* < 0.01, *P* < 0.001, and *P* < 0.0001, respectively.

## Supporting Information Available

Supplementary methods, NMR results of the polymers, results of the swelling assay of iCPC@MgO composite hydrogel, hydrogel degradation and rheological tests, the dimeric structure of the PXS polymer, biocompatibility tests with HGFs, live/dead staining quantification results for hPDLSCs, scratch assay for hPDLSCs and HGFs, TEM images of P. gingivalis treated with hydrogel, rat periodontitis model images, systemic toxicity assessment, and primer sequences and antibodies used in the study (PDF)
iCPC copolymer (MP4)
PXS polymer (MP4)
Raw data of GPC for PXS (PDF)
