# Supplementary material for: Multifunctional Injectable Bioadhesive with Toll-like Receptor 4 and Myeloid Differentiation Factor 2 Antagonistic Anti-inflammatory Potential for Periodontal Regeneration
Source: ACS Nano. 2025 Feb 14;19(7):7098–116. doi: 10.1021/acsnano.4c15922 (PMC11867008; doi:10.1021/acsnano.4c15922)
Supplement: Supplementary file 1 — nn4c15922_si_001.pdf [file nn4c15922_si_001.pdf]

Supporting Information

Multifunctional Injectable Bioadhesive with Toll-like receptor 4 and myeloid differentiation factor 2 Antagonistic Anti-Inflammatory Potential for Periodontal Regeneration

Shuting Gao[a], Huihua Li[a], Zekun Li[b], Hong Wang[a], Xinyue Li[c], Shengyan Yang[a], Lin Huang[a], Baoping Zhang[d], Kailiang Zhang[d], James Kit Hon Tsoi[a], Jian He[b], Waruna Lakmal Dissanayaka[a*]

[a] *Applied Oral Sciences & Community Dental Care, Faculty of Dentistry, The University of Hong Kong, Hong Kong SAR.*

[b] *Department of Chemistry, Faculty of Science, The University of Hong Kong, Hong Kong SAR.*

[c] *State Key Laboratory of Applied Organic Chemistry and Key Laboratory of Nonferrous Metal Chemistry and Resources Utilization of Gansu Province, Lanzhou University, Lanzhou 730000, China.*

[d] *Department of Stomatology Lanzhou University, Lanzhou University, Lanzhou 730000, China.*

1. Supplementary methods

1.1. iCPC@MgO composite hydrogels validation

1.1.1. Swelling ratio and degradation profiles of iCPC@MgO composite hydrogel

The initial dried hydrogel was weighted as W_0. The hydrogel was then immersed in distilled water at 2, 4, 8, 12, 24 and 48 hours. After each immersion period, the swollen hydrogel was removed from the water, and the excess water was blotted off to record the weight as W_d. The swelling ratio could be calculated using Eq. (A). To evaluate the degradation of the iCPC@MgO composite hydrogel, the dried hydrogel specimens were accurately weighed (W_0) and soaked in PBS solution at 37°C in shaking for 2, 4, 7, 14, and 21 days. The remaining hydrogel was freeze-dried after discharging the PBS at the prescribed time to obtain the residual mass (W_t). The degradation ratio was calculated using Eq. (B) respectively :[1]

$$\text{Swelling ratio (\%)} = \frac{Wd}{W0} \times 100\% \quad \text{(A)}$$

$$\text{Mass loss (\%)} = \frac{W0-Wt}{W0} \times 100\% \quad \text{(B)}$$

1.1.2. Soft tissue adhesive strength of iCPC copolymer and iCPC@MgO composite hydrogel under saliva conditions

The adhesion strengths of iCPC copolymer and iCPC@MgO composite hydrogel were investigated by tensile-stress and lap shear experiments using a universal testing machine under a 500 N load cell with stretch rate of 1cm min^{-1} at room temperature. Fresh porcine skin was cut into a 4 mm × 4 mm section, then cemented on the surface of a 'T' shaped sheet. The tissues were wetted by soaking in artificial saliva before use. 500 μL of the iCPC copolymer and iCPC@MgO composite hydrogel were added and spread over the surface of tissues; the two strips were brought in contact and allowed to cure for 5 minutes. Each sample was tested five times in parallel.

1.1.3. Rheological measurements

The iCPC@MgO composite hydrogel and hydrogel's pre-solution were measured by using a dynamic rheometer (TA Instrument, USA) equipped with a parallel plate

(diameter = 40 mm, gap =50 μm). At least triplicates were carried out for each rheological measurement.

1.2. Computational molecular docking with MD2 protein

Computational molecular docking was used to compare PXS polymers with the known TLR4 antagonist Eritoran;[2] the MD2 protein was set as the docking target. The molecular dynamics simulation time was set to 100 ns. The specific steps followed were based on previously published studies and included the following steps:[3,4]

1) Preparation of ligands and receptors

The protein structure file for TLR4-MD2 (PDB ID: 2E56) was downloaded from the Protein Data Bank (www1.rcsb.org) database. The PyMOL 2.5.4 software was used to remove crystal water and co-crystallized ligands from the protein structure. The Auto Dock Tools 1.5.6 software was used to perform hydrogenation on the protein and set the docking range parameters using the Grid module (docking range referenced from the site where the co-crystallized ligand is located in the protein, Center (36.3, 24.5, 4.4), Size (29.0×22.0×21.0).

2) Molecular docking calculations and methodological validation

Poly xylitol succinate (PXS) degree of polymerization was obtained as about 2 based on the molecular weight (Mn) of PXS was calculated to be 687 kDa. The Auto Dock Vina 1.2.0 software performed molecular docking calculations on the protein and small molecules separately. The exhaustiveness parameter was set to 25 for calculation accuracy. The algorithm used was the Lamarckian Genetic Algorithm, and the docking mode was set to semi-flexible docking. Methodological validation was performed using the co-crystallized ligand in MD2, where an RMSD value of less than 2 Å between the ligand before and after docking represents successful methodological validation.

3) Molecular dynamics simulation

Molecular docking employs semi-flexible docking, which currently cannot consider the flexibility of the protein structure. To further demonstrate the degree and stability of binding between the ligand and protein, this study performed a 100 ns molecular dynamics simulation on MD2 protein alone, MD2 with the co-crystallized ligand, and

the ligand itself.

The Gromacs 2022.4 package was used to perform a 100 ns molecular dynamics simulation on the protein-ligand complexes obtained from molecular docking. The Amber14sb force field and Gaff2 force field were used for the protein and ligand, respectively. The SPC/E water model was added to the system, and a periodic boundary with a box size of 1.2 nm was established. The particle mesh Ewald (PME) method was used to calculate long-range electrostatic interactions. Monte Carlo ion placement was used to introduce an appropriate number of sodium and chloride ions to neutralize the entire system's charge.

Before the actual simulation, the system underwent three steps of energy minimization and equilibration: (1) Energy minimization of each system using a steepest descent algorithm with 50,000 steps (Stop minimization when the maximum force <1000 kJ/mol). (2) NVT ensemble equilibration of each system with constant particle number, volume, and temperature (310K) for 50,000 steps with a time step of 2 fs. (3) NPT ensemble equilibration of the entire system with constant particle number, pressure (atmospheric pressure), and temperature (310K) for 50,000 steps with a time step of 2 fs. After energy minimization and equilibration, a 100 ns molecular dynamics simulation was performed without any constraints using a time step of 2 fs, and the structure coordinates were saved every 10 ps. The resulting trajectories from the molecular dynamics simulations of the nine complexes were analyzed for root mean square deviation (RMSD), root mean square fluctuation (RMSF), radius of gyration (Rg), hydrogen bond count between the protein and compound in the complex, relative free energy distribution, and binding free energy between the protein and ligand using the MM/GBSA method.

1.3. Anti-inflammatory properties evaluations

1.3.1. Simulation of the inflammatory condition by lipopolysaccharide (LPS-p.g.)

This procedure was performed as described previously.[5] HGFs were cultured in DMEM supplemented with 10% FBS, 1% penicillin/streptomycin, and fibroblast growth supplement under 5% CO_2, and 37 °C. Once attached to the culture dish, HGFs were

cultured in a medium supplemented with 2% FBS and 1 μg mL^{-1} LPS for 6, 12, and 24 hours to stimulate the inflammatory status.

1.3.2. IL-6 level assessment by ELISA for polymers and hydrogel

To compare the anti-inflammatory effects of materials under different conditions, we divided the samples into LPS-treated and untreated groups. HGFs were pretreated with 1 μg mL^{-1} LPS for 24 hours to simulate a high inflammation state, then washed three times with PBS and added a cultural medium containing PXS polymer (2.16 mg mL^{-1}), iCPC copolymer (2.7 mg mL^{-1}), and iCPC@MgO hydrogel (4 mg mL^{-1}) for 6, 12, and 24 hours. The LPS-treated group served as the control. For the LPS-untreated group, cells with no treatment served as the control, while the experimental groups were the same as mentioned above. The culture media were collected and centrifuged to remove cell debris and used for ELISA of IL-6. Briefly, a 96-well plate pre-coated with an anti-IL-6 antibody was utilized, where culture media and standards were added to the wells and incubated to allow IL-6 binding for 2 hours. Following washing steps by PBS, a biotinylated secondary antibody was added, followed by streptavidin-HRP conjugate incubation. The results were quantified using a microplate reader at 450 nm, and the concentration was calculated using a standard curve.

1.3.3. qRT-PCR evaluation for nuclear factor-κB (NF-κB) pathway

The conditions and groups were identical to those described under 1.2.2 above. After 24 hours of treatment, cells were harvested for total RNA extraction to evaluate the gene expression of IL-6, NF-κB inhibitor α (IκBα), p65 (RelA), p50 (NFκB1), IκB Kinase α (IKKα) and Arid 5a (primer listed in TableS1). The PCR steps were consistent with those mentioned in 2.9 (Materials and methods).

1.4. Antibacterial properties of iCPC@MgO hydrogel in vitro

1.4.1 Optical density at 600 nm (OD_{600})

The anti-bacterial properties of hydrogel were tested against *Porphyromonas gingivalis* (*P.g*) ATCC 33277 and *Aggregatibacter actinomycetemcomitans (A.a)*

ATCC 29522, which were cultured in BHI medium at 37 °C under anaerobic conditions. The concentration of hydrogel was diluted into 4 gradient concentrations by culture medium: 4, 2, 1, 0.5, and 0.25 μg mL^{-1}, respectively for the following assays. The control group was treated with PBS.

OD_{600} was used to estimate the concentration of bacterial cells in a culture with hydrogel at different concentrations. 50 μL of bacteria suspension (5×10^5 mL^{-1}) and 150 μL medium containing different concentrations of hydrogel (as mentioned above) were added to 96-well plates, cultured at 37 °C for 8 hours, and the optical density was measured and recorded at 600 nm every 1 hour via microplate reader (ThermoFisher, USA).

1.4.2. Colony forming unit (CFU) counts

CFU counts were used to assess the growth kinetics of the bacterial colony forming. 10^5 CFU of *P.g* and *A.a* bacterial suspensions were treated with PBS and 4 mg mL^{-1} concentrations of hydrogel receptively. After 4 hours of incubation at 37 °C, the above bacterial solution was diluted 10^2 times with a culture medium. Then the diluted bacterial solution (100 μL) was spread onto agar plates or blood agar plates, and incubated for 24 hours, and the colonies were observed. The colony-forming units at 3× per replicate were imaged by the Zeiss Axio Observer A1 and quantified by ImageJ software.

1.4.3. Live/dead bacteria staining assay

To evaluate the bactericidal efficacy of the hydrogel, live/Dead bacteria staining assay was performed. 150 μL of 4 mg mL^{-1} hydrogel and 50 uL of bacterial suspension were added to a 96-well microplate. After 24 hours of incubation at 37 °C, the bacteria were collected by centrifugation at 10,000 × g for 10 minutes. Then, the supernatant was removed, and the bacteria was resuspended with 2 mL of 0.85% NaCl buffer. The bacteria were stained by 3 μM green-fluorescent nucleic acid dye (SYTO 9) and 30 μM red-fluorescent nuclear and chromosome dye, Propidium Iodide (PI) for 30 minutes at room temperature, then 10 uL of bacterial suspension was detected on a glass slide by

CLSM (ZEISS LSM 900, Germany). For SYTO 9 detection, the excitation wavelength was 488 nm and the emission wavelength was 525 nm. For PI detection, the excitation wavelength was 535 nm and the emission wavelength was 615 nm. Live/Dead staining results were quantified using ImageJ by thresholding the fluorescence images and measuring the fluorescence intensities.

1.4.4. Biofilm formation inhibition

The inhibition of biofilm formation can effectively reflect the antibacterial efficacy of the hydrogel. The bacterial suspension of *P.g* and *A.a* (2×10^8 mL^{-1}) was added to the confocal dish and incubated at 37 °C with 4 mg mL^{-1} of hydrogel. The cultural medium was replaced every 3 days, and the dishes were carefully washed three times with PBS to remove unadhered bacteria. After 7 days of incubation, the resulting biofilms were rinsed three times with PBS to remove planktonic bacteria. Biofilms were stained with SYTO™ 9 (green fluorescence) for 30 minutes and observed on a confocal laser scanning microscope using a 488 nm laser as excitation light with a z-axis. The ImageJ software was used to reconstruct the 3D images of biofilms, and the biofilm biomass was analyzed by COMSTAT 2 based on green fluorescence.[6]

1.4.5. Transmission electron microscopy (TEM)

TEM was conducted to observe the microstructural changes of *P.g*. The bacterial suspension of *P.g* (2×10^8 mL^{-1}) was added to the 6-well plates and incubated at 37 °C with 4 mg mL^{-1} concentration of hydrogel. After 24 hours, the bacteria precipitation was obtained by centrifugation at 10,000 rpm for 10 minutes. Then, the bacteria were fixed with 2.5% glutaraldehyde at 4 °C overnight. The samples were washed by PBS three times and dehydrated using gradient alcohol (50, 70, 90, 100%), then embedded in resin, sectioned, stained, and observed under a Transmission electron microscope (JEM 1400 PLUS, USA).

1.5. Periodontitis model establishment

Eighteen male Sprague-Dawley (SD) rats (8 weeks old, 400-450 g) were obtained from

The Laboratory Animal Organization, Lanzhou University, and used to establish a bilateral molar periodontitis model according to the previous studies.[7] Briefly, a 2-0 silk ligature was tied around the maxillary second molar of rats for 2 weeks and the *P.g* bacterial suspension was inoculated to the ligature silk every three days to induce periodontitis. The establishment the periodontitis model was confirmed by the clinical diagnostic criteria, including bleeding on probing, alveolar bone resorption, and significant bone height reduction.[8] All rats were randomly divided into 3 groups: (1) Periodontitis treated with PBS (PD-PBS); (2) Periodontitis treated with traditional commercial minocycline hydrochloride gel (PD-Minocycline); (3) Periodontitis treated with iCPC@MgO hydrogel (PD-Hydrogel). After removing the ligature silk, the treatment process was followed by approximately 0.2 mL of gel and PBS were injected into the periodontal pocket each time, once every three days, with the treatment lasting for one month. After 4 weeks, the rats were euthanized using 90% volume per minute of carbon dioxide for 10 minutes; the maxillary bone was collected and fixed in 4% paraformaldehyde for the following assays.

2. Supplementary Results:

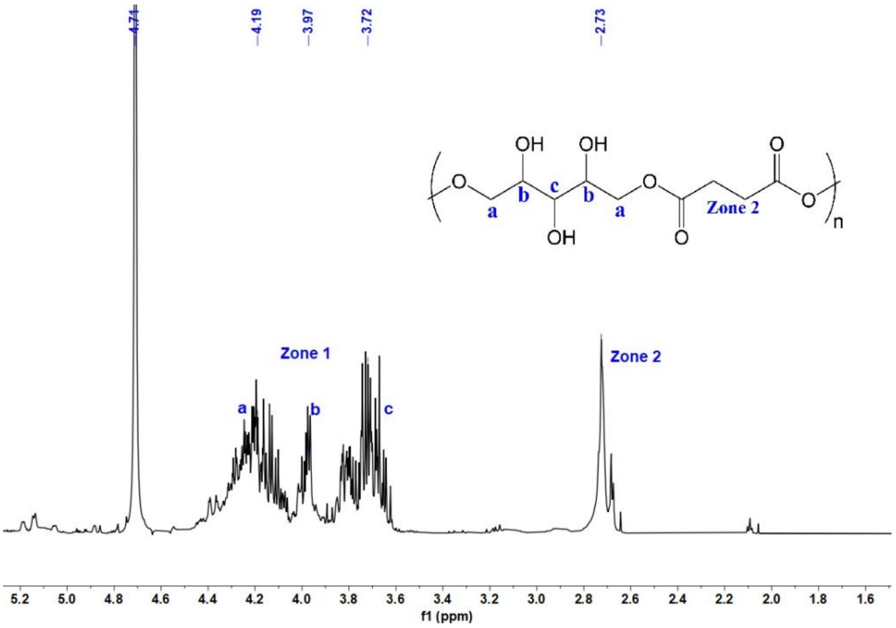

Fig. S1. Spectroscopy characterization of *poly (xylitol succinate)* (PXS polymer) derivatives.

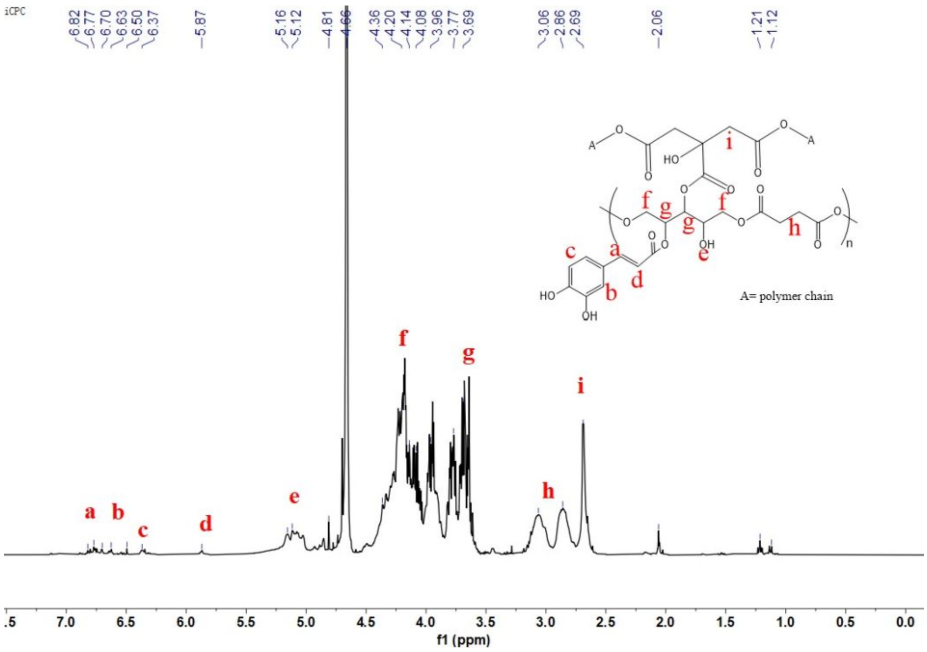

Fig. S2. Spectroscopy characterization of citric acid-*poly (xylitol succinate)*-caffeic acid (iCPC copolymer) copolymer derivatives.

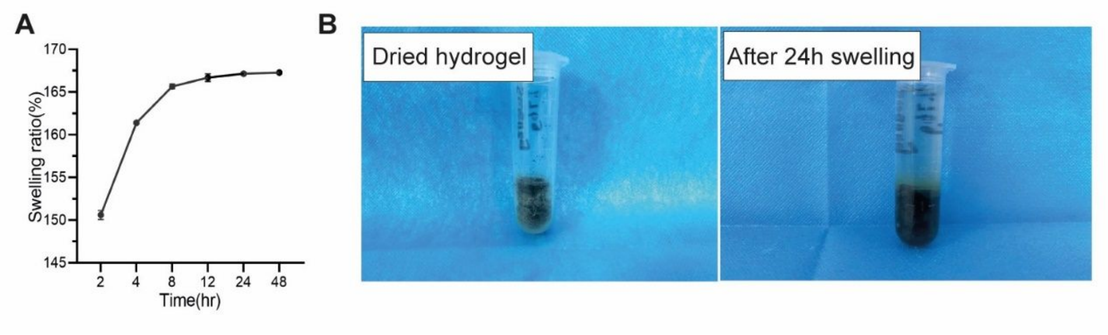

Fig. S3. The swelling assay of iCPC@MgO composite hydrogel. The maximum swelling ratio of the hydrogel reached about 167% of its weight, and the swelling plateaued from 12 hours onward (A). After lyophilized, the iCPC@MgO hydrogel exhibits a porous structure (Left). The image on the right shows the state of the hydrogel after swelling for 24 hours (B).

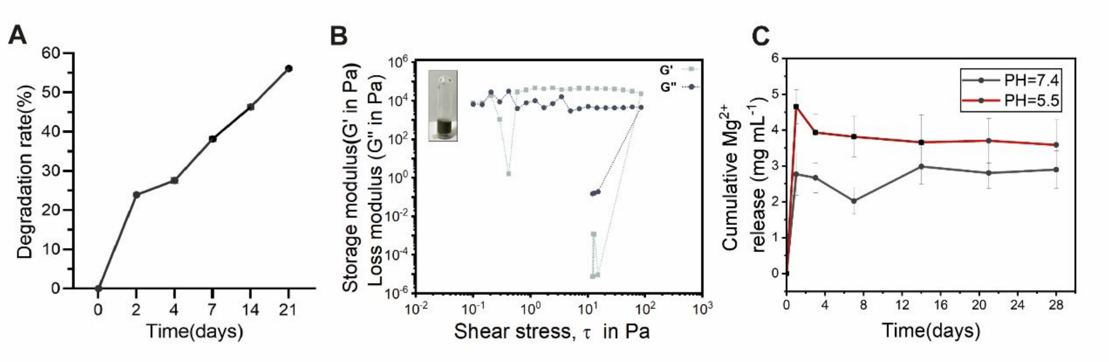

Fig. S4. Synthesis and characterization of the injectable bioadhesive iCPC@MgO composite hydrogel. (A) Hydrogel degradation rate over 21 days. (B) Rheological testing for pre-solution of iCPC@MgO composite hydrogel before ultrasound mixing. (C) Slow-release effect of Mg^{2+} at different pH conditions over 4 weeks.

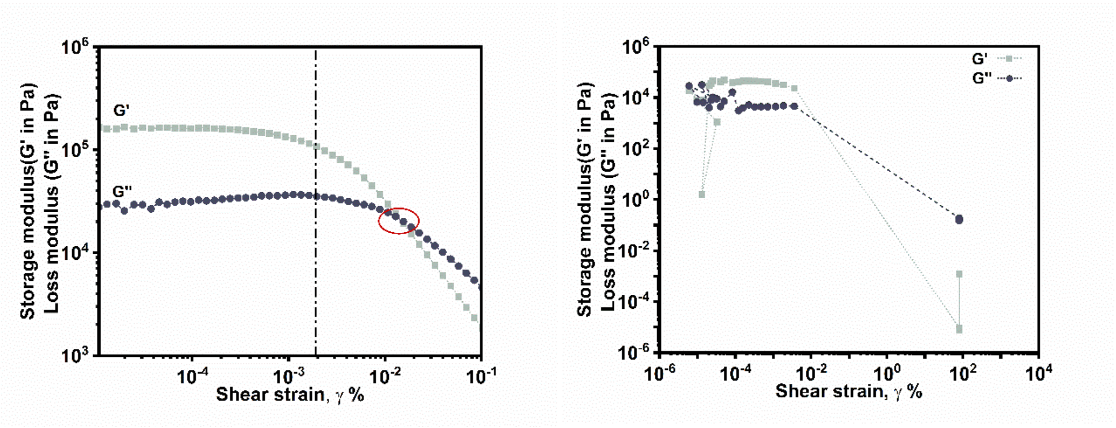

Fig. S5. Rheological properties of iCPC/MgO hydrogel after ultrasound mixing (Right) versus the mixed solution before ultrasound crosslinking (Left). The rheological properties were evaluated by plotting the storage modulus (G') and loss modulus (G'') against shear strain. The results showed that the storage modulus remained higher than the loss modulus across the tested range in iCPC@MgO hydrogel, indicating the hydrogel's predominant elastic behavior compared with the iCPC/MgO mix solution. These results indicated that ultrasound is an effective way to crosslink iCPC copolymer with MgO.

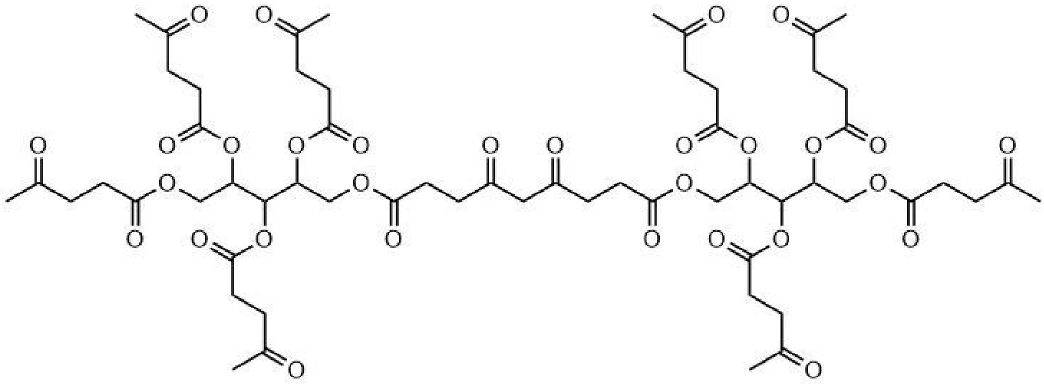

Fig. S6. The dimeric structure of the PXS polymer applied in molecular docking studies.

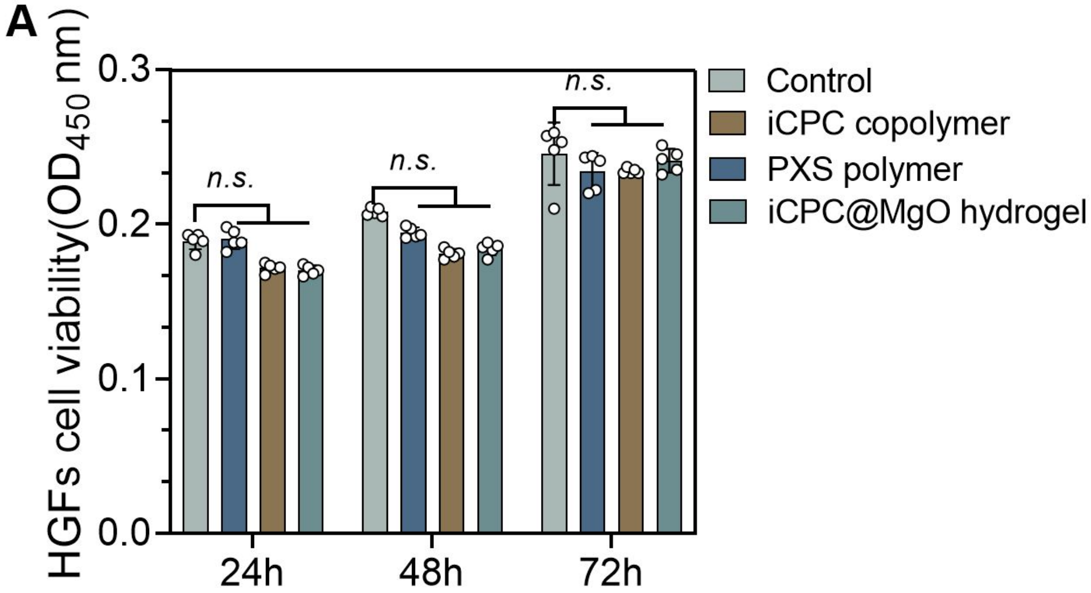

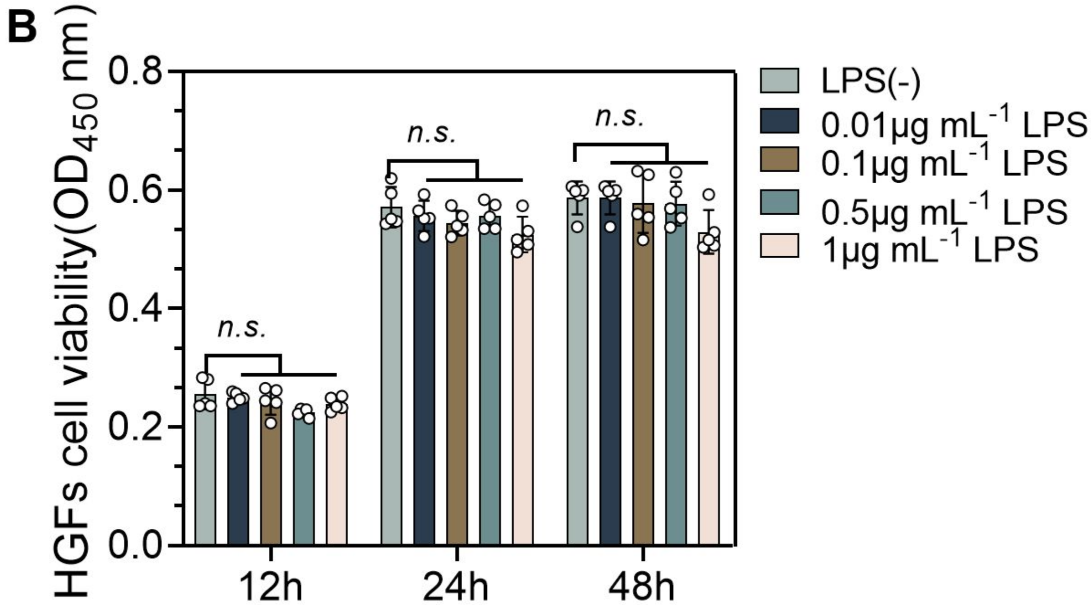

Fig. S7. Cytotoxicity evaluations of human gingival fibroblasts (HGFs) after incubation with the polymers and hydrogel for 24, 48, and 72 hours (A). Cell viability values of HGFs after being treated with different concentrations of lipopolysaccharide (LPS) at 12, 24, and 48 hours (B). Data are presented as mean ± s.d. Statistical significance was analyzed using one-way ANOVA (*$p < 0.05$, **$p < 0.01$, ***$p < 0.001$, *n.s.* indicates nonsignificance).

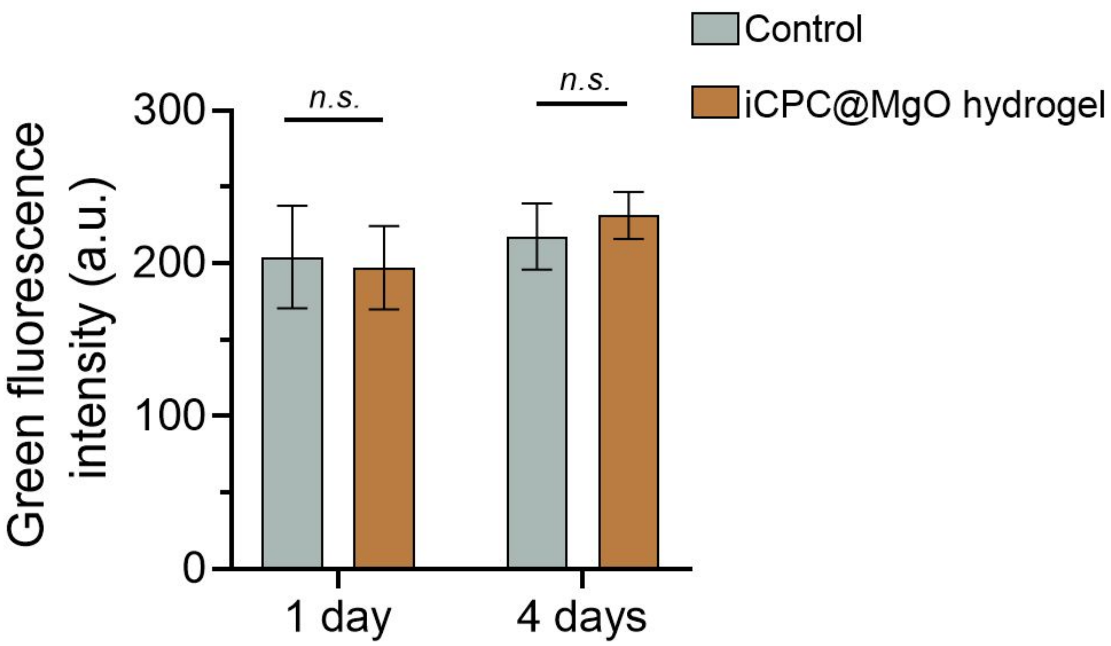

Fig. S8. Live/dead staining quantification results for hPDLSCs at 1 and 4 days. Green fluorescence represents live cells (A). Data are presented as mean ± s.d. Statistical significance was analyzed using one-way ANOVA (*$p < 0.05$, **$p < 0.01$, ***$p < 0.001$, *n.s.* indicates nonsignificance).

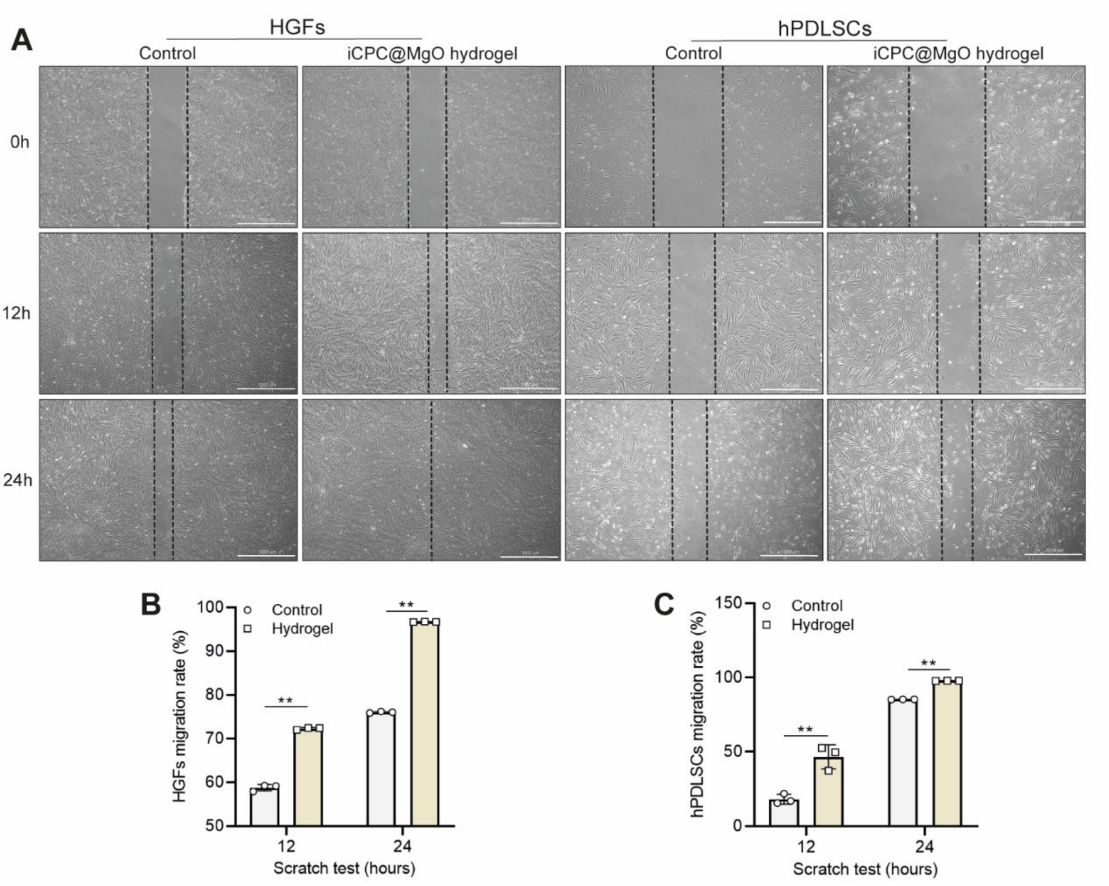

Fig. S9. The scratch assay of periodontal ligament stem cells (hPDLSCs) and gingival fibroblasts (HGFs). (A) Optical micrograph images (4X) of the scratch assay after co-culture with 4 mg mL^{-1} iCPC@MgO hydrogel for 12 and 24 hours. Quantitative analysis of migration rates indicates that the hydrogel promoted the migration of HGFs (B) and hPDLSCs (C). Data are presented as mean ± s.d. Statistical significance was analyzed using one-way ANOVA (*$p < 0.05$, **$p < 0.01$, ***$p < 0.001$, n.s. indicates nonsignificance).

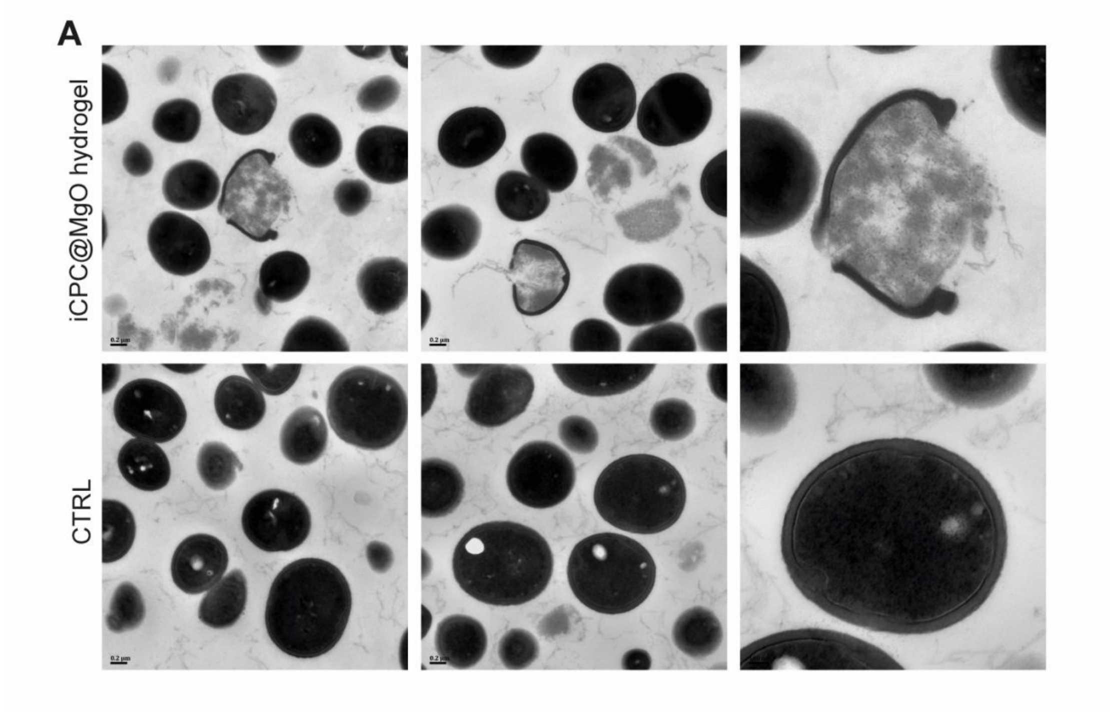

Fig. S10. Transmission electron microscopy (TEM) images of *P.g* bacteria after being treated with 4 mg mL^{-1} hydrogel for 24 hours.

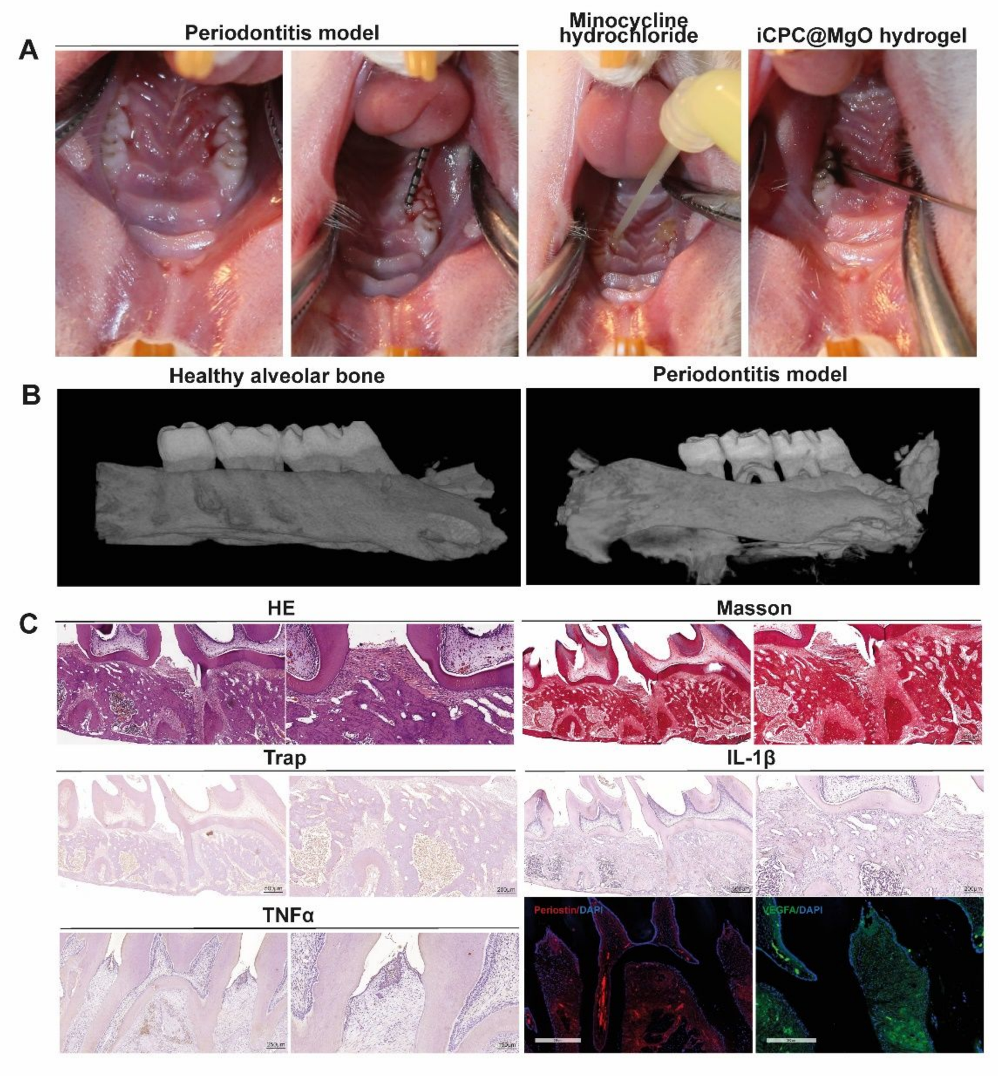

Fig. S11. The periodontal examination after inducing periodontitis revealed gingival bleeding on probing and swelling. The images show the treatment procedure for injecting the hydrogel (A) and the Micro CT images of the healthy alveolar bone vs alveolar bone affected by induced periodontitis (B). The different histological images of the minocycline hydrochloride group (C).

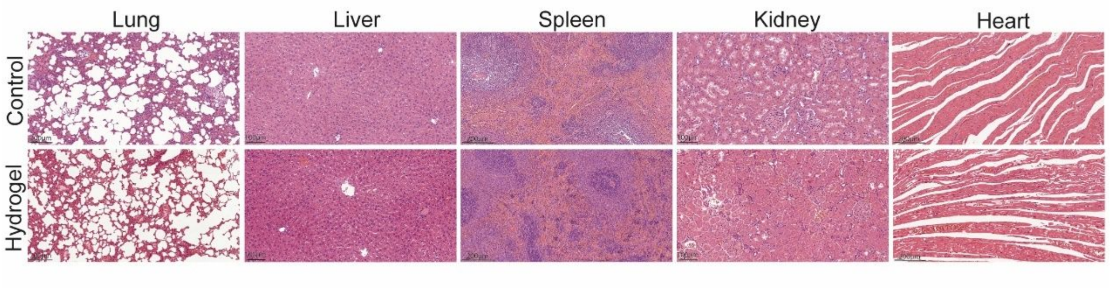

Fig. S12. Systemic toxicity assessment. The H&E staining results of the lungs, liver, spleen, kidneys, and heart were compared between the hydrogel-treated and control groups.

Table S1. Primer sequences and target regions of the nine primer pairs used in the current study.

Gene Name (Human)	Primer Sequence (5’ to 3’)
**β-catenin**	Forward AAAGCGGCTGTTAGTCACTGG
	Reverse CGAGTCATTGCATACTGTCCAT
**Runx2**	Forward AGATGATGACACTGCCACCTCTG
	Reverse GGGATGAAATGCTTGGGAACT
**Gsk 3β**	Forward GGCAGCATGAAAGTTAGCAGA
	Reverse GGCGACCAGTTCTCCTGAATC
**IL-6**	Forward ACTCACCTCTTCAGAACGAATTG
	Reverse CCATCTTTGGAAGGTTCAGGTTG
**IκBα**	Forward CTCCGAGACTTTCGAGGAAATAC
	Reverse GCCATTGTAGTTGGTAGCCTTCA
**p65**	Forward ATGTGGAGATCATTGAGCAGC
	Reverse CCTGGTCCTGTGTAGCCATT
**p50**	Forward AACAGAGAGGATTTCGTTTCCG
	Reverse TTTGACCTGAGGGTAAGACTTCT
**IKKα**	Forward GGCTTCGGGAACGTCTGTC
	Reverse TTTGGTACTTAGCTCTAGGCGA
**Arid 5a**	Forward CCCCAACGTCCTTCGACAG
	Reverse CTGCATAGGTTGTGACTGGTG

Table S2. Detailed information on antibodies used in the current study.

Antibody	Origin species	Target	Dilution	Cat. No. and supplier
**β catenin**	Rabbit	Anti-human	1:1000	47993 Signalway Antibody
**Runx2**	Rabbit	Anti-human	1:1000	41746 Signalway Antibody
**Gsk 3β**	Rabbit	Anti-human	1:1000	40989 Signalway Antibody
**IL-1β**	Rabbit	Anti-Rat	1:100	16806-1-AP Proteintech
**TNF-α**	Rabbit	Anti-Rat	1:100	17590-1-AP Proteintech
**TRAP**	Rabbit	Anti-Rat	1:100	ab191406 Abcam
**Periostin**	Rabbit	Anti-Rat	1:100	19899-1-AP Proteintech
**IgG H&L secondary antibody (Alexa Fluor® 647)**	Goat	Anti-Rabbit	1:1000	ab150091 Abcam
**IgG H&L (HRP) secondary antibody**	Goat	Anti-Rabbit	1:1000	ab6721 Abcam

3. References

(1) Grindy, S.C.; Learsch, R.; Mozhdehi, D.; Cheng, J.; Barrett, D.G.; Guan, Z.; Messersmith, P.B.; Holten-Andersen, N. Control of Hierarchical Polymer Mechanics with Bioinspired Metal-Coordination Dynamics *Nat Mater.* **2015** *14(12)*, 1210-6.

(2) Opal, S.M.; Laterre Pf Fau - Francois, B. et al. Effect of Eritoran, an Antagonist of MD2-TLR4, on Mortality in Patients with Severe Sepsis: the ACCESS Randomized Trial, *JAMA*.**2013**, *309*, 1154-1162.

(3) Bender, B.J.; Gahbauer, S.; Luttens, A.; Lyu, J.; Webb, C.M.; Stein, R.M.; Fink, E.A.; Balius, T.E.; Carlsson, J.; Irwin, J.J.; Shoichet, B.K. A Practical Guide to Large-Scale Docking. *Nat Protoc*. **2021**, *16*, 4799-4832.

(4) Agu, P.C.; Afiukwa, C.A.; Orji, O.U.; Ezeh, E.M.; Ofoke, I.H.; Ogbu, C.O.; Ugwuja, E.I.; Aja, P.M. Molecular Docking as a Tool for the Discovery of Molecular Targets of Nutraceuticals in Diseases Management. Sci. Rep. **2023**, *13(1)*, 13398.

(5) Herath, T.D.K.; Wang, Y.; Seneviratne, C.J.; Lu, Q.; Darveau, R.P.; Wang, C.-Y.; Jin, L. Porphyromonas Gingivalis Lipopolysaccharide Lipid A Heterogeneity Differentially Modulates the Expression of IL-6 and IL-8 in Human Gingival Fibroblasts. J. Clin. Periodontol. **2011**, *38(8)*, 694–701.

(6) Shi, Y.; Cao, Y.; Cheng, J.; Yu, W.; Liu, M.; Yin, J.; Huang, C.; Liang, X.; Zhou, H.; Liu, H.; Yang, Z.; Fang, Y.; Wei, H.; Zhao, G. Construction of Self-Activated Nanoreactors for Cascade Catalytic Anti-Biofilm Therapy Based on H2O2 Self-Generation and Switch-On NO Release. Adv. Func. Mater. **2022**, *32(20)*, 2111148.

(7) Marchesan, J.; Girnary, M.S.; Jing, L.; Miao, M.Z.; Zhang, S.; Sun, L.; Morelli, T.; Schoenfisch, M.H.; Inohara, N.; Offenbacher, S.; Jiao, Y. An Experimental Murine Model to Study Periodontitis. Nat. Protoc. **2018**, 2247–2267.

(8) Tonetti, M.S. Treatment of Stage I–III Periodontitis—The EFP S3 Level Clinical Practice Guideline. J. Clin. Periodontol. **2020**, *47(S22)*, 4–60.
