# Supplementary material for: Multifunctional Injectable Bioadhesive with Toll-like Receptor 4 and Myeloid Differentiation Factor 2 Antagonistic Anti-inflammatory Potential for Periodontal Regeneration
Source: ACS Nano. 2025 Feb 14;19(7):7098–116. doi: 10.1021/acsnano.4c15922 (PMC11867008; doi:10.1021/acsnano.4c15922)
Supplement: Supplementary file 4 — nn4c15922_si_005.pdf [file nn4c15922_si_005.pdf]

Project Name: 20201203
Reported by User: Breeze

Breeze 2 HPLC System

SAMPLE INFORMATION

Sample Name:	KOUQIANG-PXS	Acquired By:	Breeze
Sample Type:	Unknown	Date Acquired:	2022/9/28 18:49:03 CST
Vial:	1:E,3	Acq. Method:	2009
Injection #:	1	Date Processed:	2022/9/29 8:58:42 CST
Injection Volume:	50.00 ul	Channel Name:	410
Run Time:	25.00 Minutes	Sample Set Name	2009

Autoscaled Chromatogram

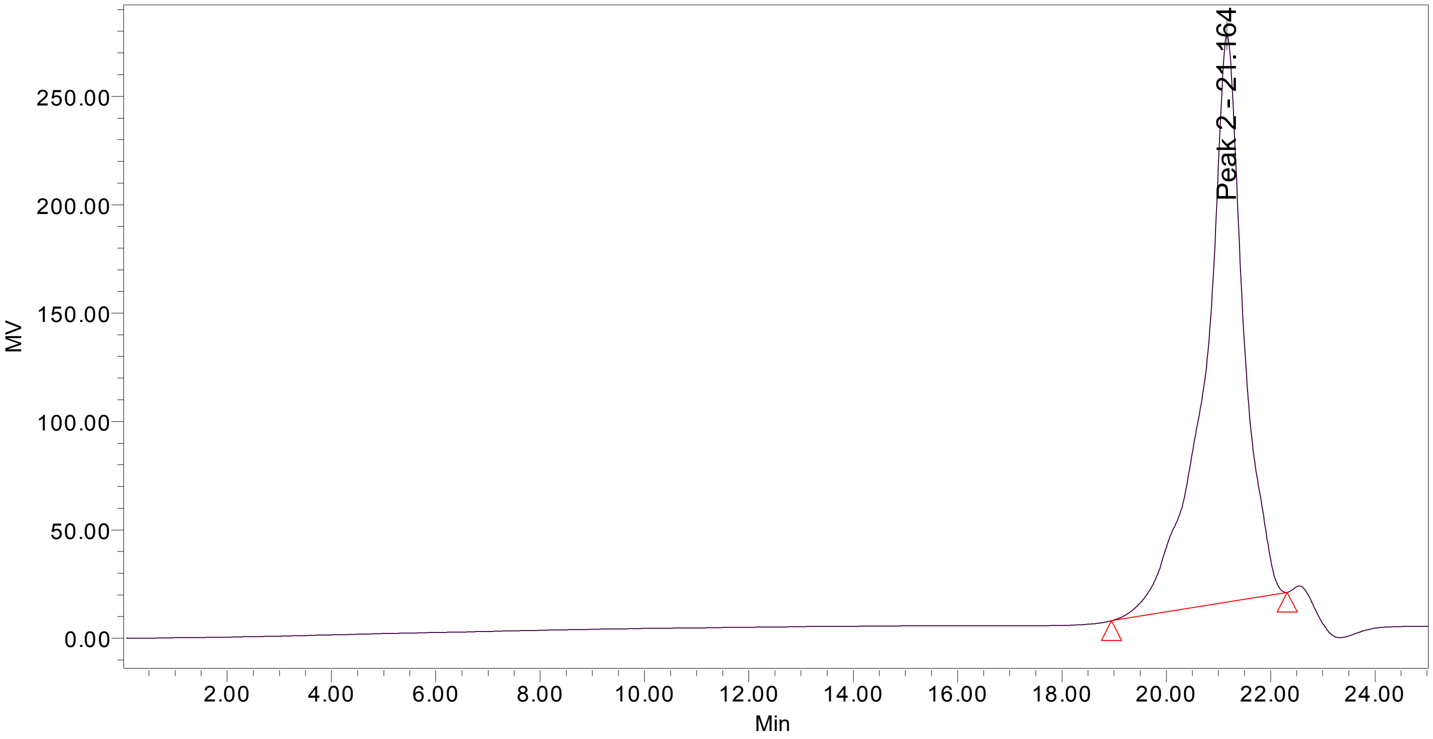

GPC Results

	Dist Name	Elution Volume (ml)	Retention Time (min)	Adjusted RT (min)	Mn	Mw	MP	Mz	Mz+1	Mz/Mw	Mz+1/Mw
1		21.164	21.164	21.164	687	697		708	721	1.016124	1.034356

GPC Results

	Area (*sec)	% Area	Height	% Height	Integration Type	Peak Codes	Points Across Peak	Start Time (min)	End Time (min)
1	13593233	100.00	261588	100.00	bb	G11	202	18.950	22.317

GPC Results

	Baseline Start (min)	Baseline End (min)	Slope (ore/sec)	Offset (ore)
1	18.950	22.317	3.890204e+000	-6.563086e+001

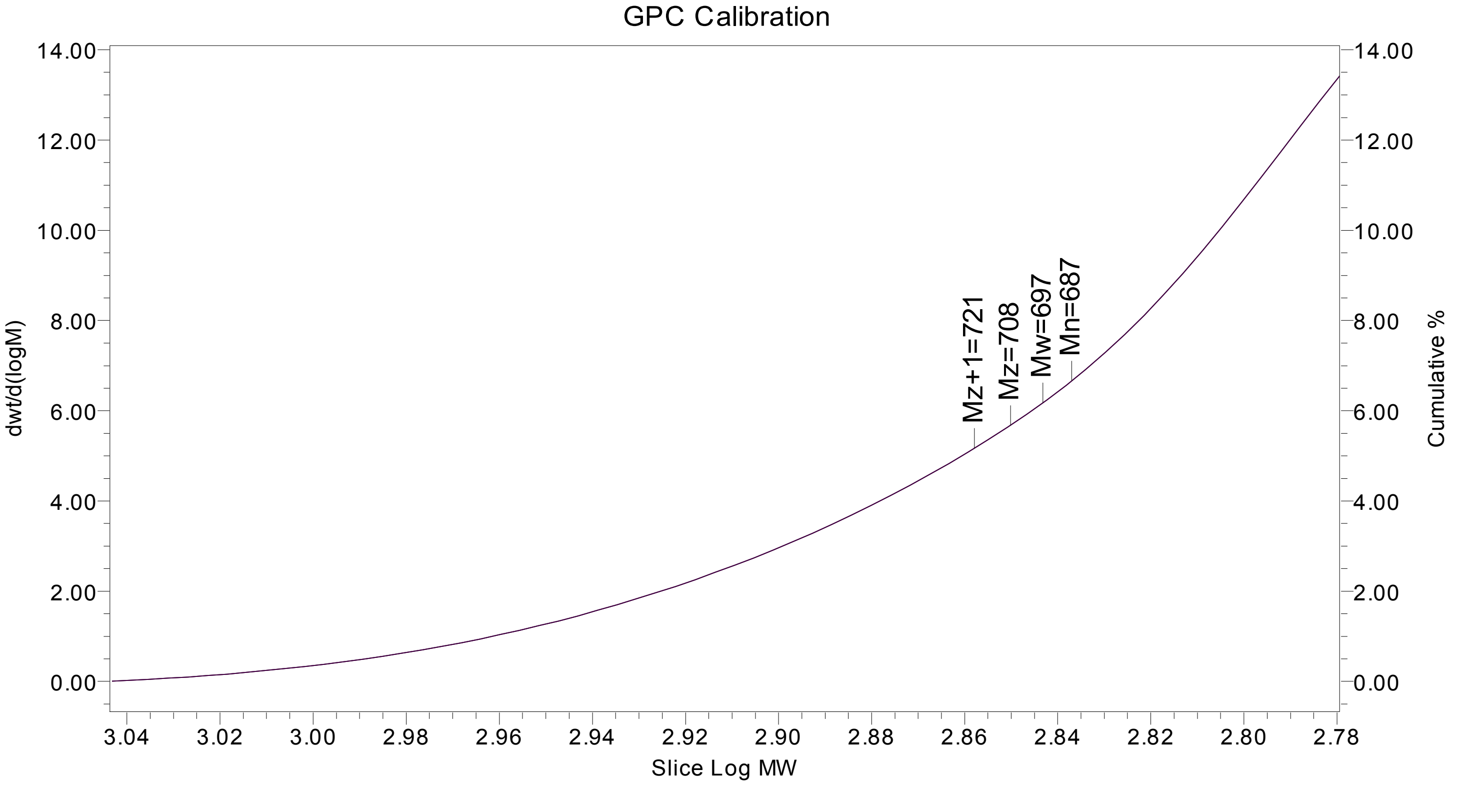

Distribution Table Mp: -50000 name: peak 2

	Slice MW (Daltons)	Slice Log MW	Slice Volume (ml)	Slice Area	dwt/d(logM)	Cumulative %	Slice [n] (dl/g)	Outside Vo-Vt
1	745	2.871953	19.633	135932	8.508259	1.000		no
2	674	2.828340	19.806	135932	14.578520	2.000		no
3	631	2.800320	19.917	135932	20.865581	3.000		no
4			20.000	135932		4.000		no
5			20.070	135932		5.000		no
6			20.132	135932		6.000		yes
7			20.189	135932		7.000		yes
8			20.243	135932		8.000		yes
9			20.293	135932		9.000		yes
10			20.339	135932		10.000		yes
11			20.381	135932		11.000		yes
12			20.420	135932		12.000		yes
13			20.455	135932		13.000		yes
14			20.488	135932		14.000		yes
15			20.519	135932		15.000		yes
16			20.548	135932		16.000		yes
17			20.576	135932		17.000		yes
18			20.603	135932		18.000		yes

Distribution Table Mp: -50000 name: peak 2

	Slice MW (Daltons)	Slice Log MW	Slice Volume (ml)	Slice Area	dwt/d(logM)	Cumulative %	Slice [n] (dl/g)	Outside Vo-Vt
19			20.629	135932		19.000		yes
20			20.654	135932		20.000		yes
21			20.678	135932		21.000		yes
22			20.702	135932		22.000		yes
23			20.724	135932		23.000		yes
24			20.745	135932		24.000		yes
25			20.766	135932		25.000		yes
26			20.786	135932		26.000		yes
27			20.805	135932		27.000		yes
28			20.824	135932		28.000		yes
29			20.841	135932		29.000		yes
30			20.858	135932		30.000		yes
31			20.874	135932		31.000		yes
32			20.890	135932		32.000		yes
33			20.904	135932		33.000		yes
34			20.919	135932		34.000		yes
35			20.932	135932		35.000		yes
36			20.945	135932		36.000		yes
37			20.958	135932		37.000		yes
38			20.970	135932		38.000		yes
39			20.982	135932		39.000		yes
40			20.993	135932		40.000		yes
41			21.004	135932		41.000		yes
42			21.015	135932		42.000		yes
43			21.025	135932		43.000		yes
44			21.035	135932		44.000		yes
45			21.045	135932		45.000		yes
46			21.055	135932		46.000		yes
47			21.065	135932		47.000		yes
48			21.074	135932		48.000		yes
49			21.083	135932		49.000		yes
50			21.092	135932		50.000		yes
51			21.101	135932		51.000		yes
52			21.110	135932		52.000		yes
53			21.119	135932		53.000		yes
54			21.128	135932		54.000		yes
55			21.136	135932		55.000		yes
56			21.145	135932		56.000		yes

Distribution Table Mp:
-50000 name: peak 2

	Slice MW (Daltons)	Slice Log MW	Slice Volume (ml)	Slice Area	dwt/d(logM)	Cumulative %	Slice [n] (dl/g)	Outside Vo-Vt
57			21.154	135932		57.000		yes
58			21.162	135932		58.000		yes
59			21.171	135932		59.000		yes
60			21.180	135932		60.000		yes
61			21.189	135932		61.000		yes
62			21.197	135932		62.000		yes
63			21.206	135932		63.000		yes
64			21.215	135932		64.000		yes
65			21.224	135932		65.000		yes
66			21.233	135932		66.000		yes
67			21.243	135932		67.000		yes
68			21.252	135932		68.000		yes
69			21.262	135932		69.000		yes
70			21.271	135932		70.000		yes
71			21.281	135932		71.000		yes
72			21.292	135932		72.000		yes
73			21.302	135932		73.000		yes
74			21.313	135932		74.000		yes
75			21.324	135932		75.000		yes
76			21.335	135932		76.000		yes
77			21.347	135932		77.000		yes
78			21.359	135932		78.000		yes
79			21.372	135932		79.000		yes
80			21.385	135932		80.000		yes
81			21.399	135932		81.000		yes
82			21.413	135932		82.000		yes
83			21.429	135932		83.000		yes
84			21.445	135932		84.000		yes
85			21.461	135932		85.000		yes
86			21.479	135932		86.000		yes
87			21.499	135932		87.000		yes
88			21.519	135932		88.000		yes
89			21.541	135932		89.000		yes
90			21.565	135932		90.000		yes
91			21.591	135932		91.000		yes
92			21.620	135932		92.000		yes
93			21.651	135932		93.000		yes
94			21.685	135932		94.000		yes

Distribution Table Mp: -50000 name: peak 2

	Slice MW (Daltons)	Slice Log MW	Slice Volume (ml)	Slice Area	dwt/d(logM)	Cumulative %	Slice [n] (dl/g)	Outside Vo-Vt
95			21.723	135932		95.000		yes
96			21.765	135932		96.000		yes
97			21.813	135932		97.000		yes
98			21.871	135932		98.000		yes
99			21.951	135932		99.000		yes
100			22.300	135932		100.000		yes
